# HP1 drives de novo 3D genome reorganization in early *Drosophila* embryos

**DOI:** 10.1038/s41586-021-03460-z

**Published:** 2021-04-14

**Authors:** Fides Zenk, Yinxiu Zhan, Pavel Kos, Eva Löser, Nazerke Atinbayeva, Melanie Schächtle, Guido Tiana, Luca Giorgetti, Nicola Iovino

**Affiliations:** 1 Max Planck Institute of Immunobiology and Epigenetics, Freiburg im Breisgau, Germany; 2 Albert-Ludwigs-Universität Freiburg, Freiburg im Breisgau, Germany; 3 Friedrich Miescher Institute for Biomedical Research, Basel, Switzerland; 4 University of Basel, Basel, Switzerland; 5 CIBSS – Centre for Integrative Biological Signalling Studies, University of Freiburg, Freiburg im Breisgau, Germany; 6 Università degli Studi di Milano and INFN, Milan, Italy

**Keywords:** Embryogenesis, Epigenetic memory, Epigenetics, Chromatin, Nuclear organization

## Abstract

Fundamental features of 3D genome organization are established de novo in the early embryo, including clustering of pericentromeric regions, the folding of chromosome arms and the segregation of chromosomes into active (A-) and inactive (B-) compartments. However, the molecular mechanisms that drive de novo organization remain unknown^1,2^. Here, by combining chromosome conformation capture (Hi-C), chromatin immunoprecipitation with high-throughput sequencing (ChIP–seq), 3D DNA fluorescence in situ hybridization (3D DNA FISH) and polymer simulations, we show that heterochromatin protein 1a (HP1a) is essential for de novo 3D genome organization during *Drosophila* early development. The binding of HP1a at pericentromeric heterochromatin is required to establish clustering of pericentromeric regions. Moreover, HP1a binding within chromosome arms is responsible for overall chromosome folding and has an important role in the formation of B-compartment regions. However, depletion of HP1a does not affect the A-compartment, which suggests that a different molecular mechanism segregates active chromosome regions. Our work identifies HP1a as an epigenetic regulator that is involved in establishing the global structure of the genome in the early embryo.

## Abstract (web summary)

The heterochromatin protein HP1 has an essential role in establishing several features of the 3D nuclear organization of the genome during early embryonic development in *Drosophila*.

## Main

In metazoans, fertilization triggers global de novo chromatin reorganization into heterochromatin and euchromatin. The clustering of pericentromeric heterochromatin and the folding of chromosome arms lead to a highly regular Rabl configuration during zygotic genome activation (ZGA)^3,4^. Concomitantly, active and inactive chromatin regions start to associate to form the A- and B-compartments, respectively^2,5–9^. The molecular determinants of compartmental forces remain unknown.

Constitutive heterochromatin is enriched for histone 3 lysine 9 di- and trimethylation (H3K9me2/3) and is important for chromatin structure^10,11^. Members of the heterochromatin protein family bind to constitutive heterochromatin and perform related functions in all eukaryotes^12^. All family members contain a chromodomain^13^, which binds to H3K9me2/3, and a chromoshadow domain, which supports homodimerization and protein–protein interactions^14^. *Drosophila* expresses five different heterochromatin protein family members^12^ termed HP1a–HP1e. HP1a (hereafter termed as HP1, encoded by *Su(var)2-5*) was discovered in *Drosophila*^15^ and is essential for early embryonic development, as is the mammalian protein HP1β^16,17^. HP1 localizes mainly to H3K9me2/3-rich heterochromatin^10,15,18^, but also to euchromatic sites along chromosome arms^19^. HP1 might promote heterochromatin compaction through phase separation^20^, similar to human HP1α^21^. Whether HP1 is required to initiate genome reorganization in early embryos is unclear.

**Fig. 1:**
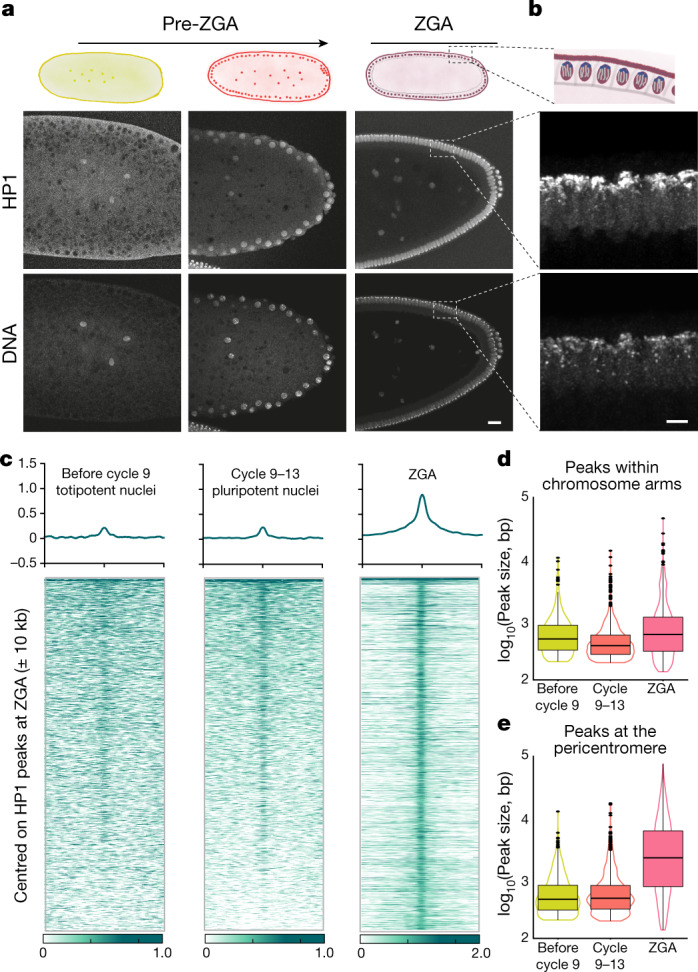
Localization of HP1 during early embryonic development. **a**, Top, schematic of early embryonic development. Bottom, immunofluorescence staining at different stages of early embryonic development. HP1 localizes to chromatin before ZGA and becomes enriched at the pericentromeric heterochromatin at ZGA. Scale bar, 20 μm. **b**, Close-up view of HP1 localization at ZGA. Top, schematic shows the Rabl configuration of the chromosomes at this developmental stage, with the centromeres localizing on top and the chromosome arms reaching to the bottom of the nucleus. Bottom, the centromeric regions display strong HP1 signals. Images in **a** and **b** are representative from four biological replicates. Scale bar, 5 μm. **c**, Heat maps of HP1 ChIP–seq signal at three different early embryonic developmental time points. The signal is centred on HP1 peaks within chromosome arms called at ZGA and ranked by signal intensity at cycles 9–13. HP1 binding to chromatin is already observed before cycle 9, and becomes more enriched during development. **d**, Box plots of HP1 peak size distribution within chromosome arms at cycle 9, cycles 9–13 and ZGA. **e**, Box plots of HP1 peak size distribution within pericentromeric regions at cycle 9, cycles 9–13 and ZGA, showing that HP1 peaks get broader at the pericentromeric regions at ZGA. In all box plots, centre line denotes the median; boxes denote lower and upper quartiles (Q1 and Q3, respectively); whiskers denote 1.5× the interquartile region (IQR) below Q1 and above Q3; points denote outliers. Source data

**Extended Data Fig. 1:**
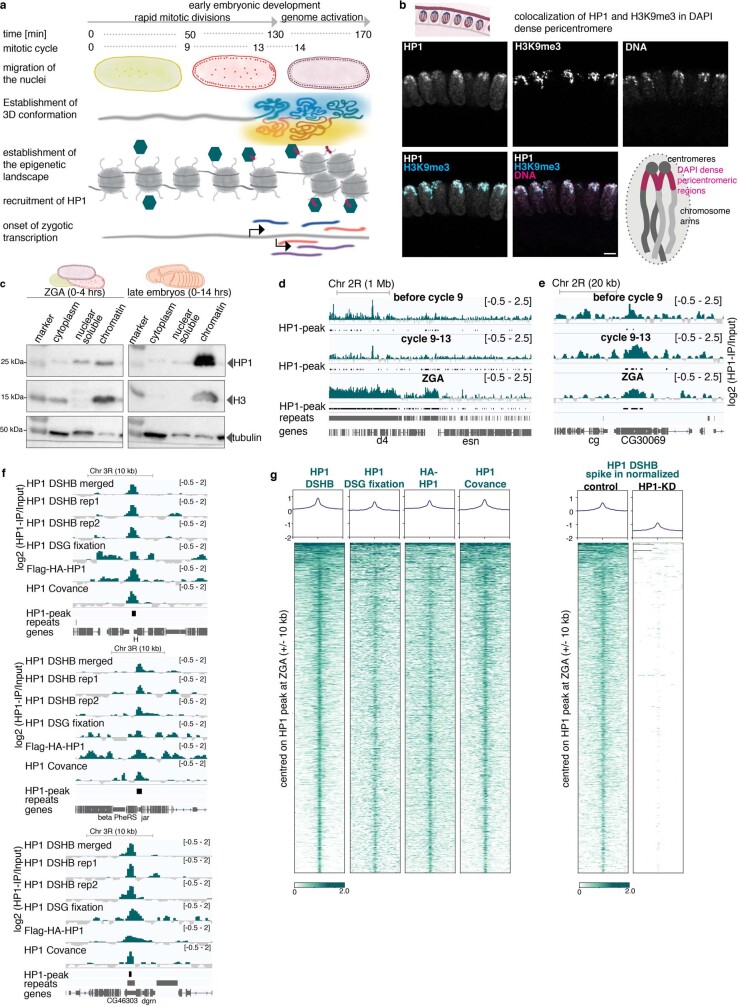
Characterization of HP1 binding during early embryonic development. **a**, Cartoon of early *Drosophila* developmental timing showing the onset of genome organization, chromatin modifications and transcription. **b**, Immunofluorescence staining of an embryo at ZGA. H3K9me3 and HP1 are enriched at the pericentromeric heterochromatin (clustering on top and corresponding to the DAPI dense signal; see cartoon). Representative image from four biological replicates. Scale bar, 5 μm. Quantification of the immunofluorescence signal shows that HP1 intensity is 30 times higher in the pericentromeric regions (co-localizing with H3K9me3) than in the rest of the nucleus. Average signal of 300 nuclei from 2 independent experiments. **c**, Cellular fractionation of embryonic extracts at 0–4 h (corresponding to ZGA) of development and from late embryos (corresponding to gastrulation and segmentation). HP1 is already detectable in the chromatin fraction at 0–4 h and becomes further enriched during differentiation. Representative of two independent experiments. For western blot source data, see Supplementary Fig. 1. **d**, **e**, Representative genomic regions showing HP1 signal as log_2_-transformed fold change over the input before cycle 9, between cycle 9–13 and at ZGA by ChIP–seq. HP1 peaks and repetitive sequences (UCSC RepeatMasker) are represented below. **d**, Strong enrichment of HP1 close to the pericentromeric heterochromatin. **e**, One euchromatic HP1 binding region. **f**, IGV browser snapshots of different genomic regions showing HP1 binding in euchromatin regions. We validated the HP1 ChIP–seq by performing replicate experiments with the same antibody from DSHB (rep1 and rep2). All further tracks in this Article show the merged track (top). To further validate our findings, we mapped the binding of HP1 by performing ChIP–seq against a Flag-haemagglutinin (HA)-tagged transgene and used a second commercial antibody (Covance) and detected the same peaks. We also used disuccinimidyl glutarate (DSG) as crosslinking agent to recover more extended regions of HP1 binding and obtained a similar result of HP1 binding. **g**, Heat maps of HP1, ChIP–seq signals ± 10 kb centred on HP1 peaks occurring along the chromosome arms at ZGA. We validated the binding profiles by performing ChIP–seq against HP1 with different antibodies (DSHB, HA, Covance) and also used the crosslinker DSG (left). To further validate the peaks within chromosome arms, we performed quantitative ChIP–seq in the HP1-KD background, using λ-DNA spike-in as normalizer. The HP1 signal is strongly reduced at HP1 peaks within chromosome arms at ZGA (right). See Supplementary Methods for further details. Source data

To address this question, we performed immunofluorescence of *Drosophila* embryos before ZGA and the establishment of higher-order chromatin architecture^5,6^, observing diffuse nuclear localization of HP1 (Fig. 1a, Extended Data Fig. 1a). By ZGA, both HP1 and H3K9me3 were strongly enriched at pericentromeric heterochromatin, which was localized apically (reflecting the Rabl configuration) and overlapped with DAPI-dense regions (Fig. 1b, Extended Data Fig. 1b, c). The HP1 signal was around 30 times higher in these regions (Supplementary Methods).

To characterize HP1 binding at different developmental stages, we performed HP1 ChIP–seq in precisely hand-staged *Drosophila* wild-type (control) embryos (Fig. 1c, Extended Data Fig. 1d, e). At ZGA, HP1 localized not only to constitutive heterochromatin, such as pericentromeric and telomeric regions (4,394 peaks, 67%) (Extended Data Fig. 1d), but also within chromosome arms (2,213 peaks, 33%) at repeat sequences (43% of non-pericentromeric peaks, 10% long interspersed nuclear elements (LINEs), 30% long-terminal repeats (LTRs)) and unique sequences (57% of peaks) (Extended Data Fig. 1d–g). Consistent with the immunofluorescence analysis (Fig. 1a), HP1 was bound to chromatin even in totipotent nuclei (Fig. 1c–e), albeit at a lower enrichment (16% of the ZGA enrichments) (Supplementary Methods). Notably, the peak size on chromosome arms did not change markedly (Fig. 1d), whereas HP1 spreading occurred at pericentromeric regions during development (Fig. 1e, Extended Data Fig. 1d, Supplementary Methods).

**Fig. 2:**
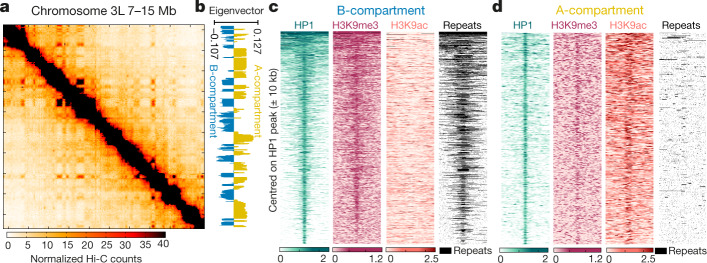
HP1 binds both A- and B-compartment regions at ZGA. **a**, Hi-C contact map of an 8-Mb region on chromosome 3L (resolution 40 kb). Pooled Hi-C data of seven biological replicates are shown (Extended Data Fig. 2a). **b**, Compartment scores (first eigenvector of the Hi-C map, resolution: 10 kb), same region as in **a** (Supplementary Methods). **c**, Heat maps of HP1, H3K9me3 and H3K9ac ChIP–seq signals as well as repeat positions, ±10 kb centred on HP1 peaks occurring in B-compartment regions. HP1 binding overlaps with broad H3K9me3 peaks, repeats and is devoid of H3K9ac. **d**, As in **c** for HP1 peaks in A-compartment regions, showing enrichment in H3K9ac and absence of repeats (Extended Data Fig. 2b–d).

**Extended Data Fig. 2:**
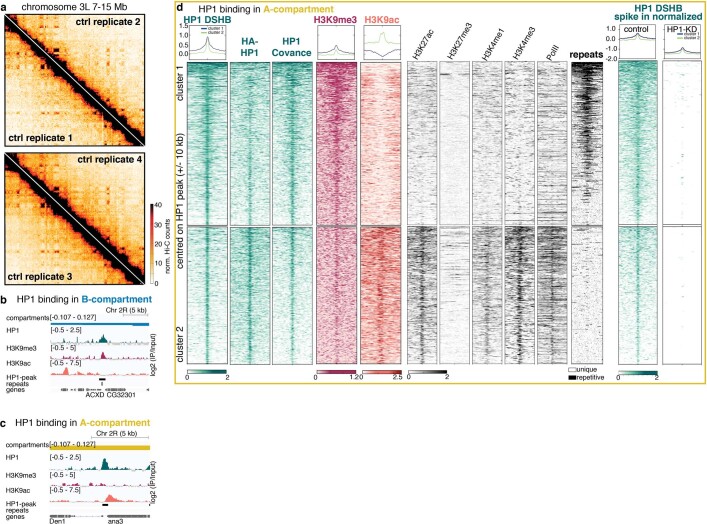
Characterization of HP1 binding within A- and B-compartment. **a**, Hi-C contact maps with contact frequencies of chromosome 3L (7–15 Mb) at a resolution of 40 kb. Four out of seven biological replicates are shown. **b**, Representative example of HP1 binding in a B-compartment region. **c**, Representative example of HP1 binding in an A-compartment region. **d**, Extended characterization of HP1 binding in A-compartment regions. The heat maps show ChIP–seq signal and repeat coordinates in ±10 kb centred around HP1 peaks. Figure 2d shows only cluster 2 containing HP1 peaks that localize within non-repetitive, active regulatory sequences enriched in H3K9ac, H3K27ac, H3K4me1/3 as well as polymerase II. We validated this cluster in active regions by performing ChIP–seq with different antibodies against HP1 (HA antibody against a Flag-HA-HP1-tagged transgene (second heat map) and HP1 Covance antibody (third heat map)). We further performed ChIP–seq in HP1-KD embryos using λ-DNA spike-in to normalize the signal and found a strong reduction of HP1 binding. This further validates the specificity of the HP1 peaks. See Supplementary Methods for further information. A second cluster of HP1 binding events (cluster 1) occurs in repetitive chromatin regions that are largely devoid of active histone modification marks. Source data

Next, we generated Hi-C data for control embryos precisely hand-staged at ZGA (Fig. 2a, Extended Data Fig. 2a). Chromosomes were clearly segregated into A- and B-compartments (Fig. 2a, b). HP1 was bound not only within B-compartment but also within A-compartment sequences (Fig. 2c, d, Extended Data Fig. 2b–d, Supplementary Methods). As expected, HP1 binding in B-compartment regions systematically overlapped with H3K9me3, localized around repeats and occasionally extended over several kilobases (median peak size 730 bp) (Fig. 2c). By contrast, we detected two different modes of HP1 binding in A-compartment regions. We found that 46% of HP1 binding sites in the A-compartment were sharply localized and enriched for active chromatin marks, and did not overlap with repeats (Fig. 2d, Extended Data Fig. 2d, cluster 2). A second class of HP1 peaks resembled those in the B-compartment (Extended Data Fig. 2d, cluster 1). These might correspond to short stretches of repetitive repressed DNA that cannot be resolved unequivocally by Hi-C. ChIP–seq analysis thus suggests that HP1 binds (1) within active, H3K9ac-rich chromatin in the A-compartment, and (2) within inactive, constitutive heterochromatic domains of the B-compartment.

**Extended Data Fig. 3:**
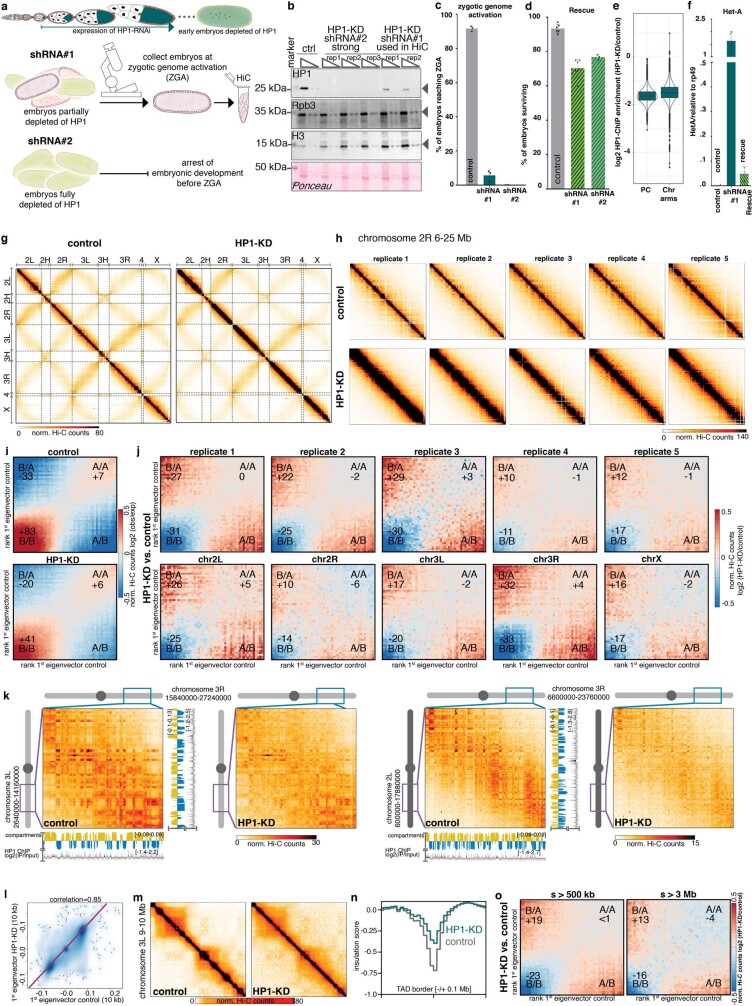
Characterization of HP1 knockdown and its effect on 3D genome organization. **a**, Schematic of the mode of action of the RNA interference (RNAi) knockdown. shRNA against HP1 is expressed only at late stages of oogenesis and does not interfere with the production of fertilized embryos. The resulting early embryo is devoid of maternally loaded mRNA and protein. The bottom part shows the two knockdowns and embryo collection strategy. **b**, Western blot showing reduction of HP1 protein in early embryos after shRNA-mediated knockdown. shRNA#1 was used to perform the Hi-C experiments and generated embryos carrying residual HP1; shRNA#2 completely depleted HP1 proteins. Rbp3, H3 and Ponceau staining were used as loading controls. Representative of two independent experiments. For western blot source data, see Supplementary Fig. 1. **c**, Following the use of shRNA#1, between 5% and 10% of the embryos reach ZGA, therefore allowing the study of 3D chromatin conformation. shRNA#2 blocked embryonic development at the first or second mitotic division, with 0% embryos reaching ZGA, therefore preventing the study of the 3D chromatin conformation establishment. Data are mean ± s.d. Number of biological replicates: 3 for control; 3 for HP1-KD shRNA#1; 3 for HP1-KD shRNA#2. **d**, Both shRNA#1 and shRNA#2 are specific towards HP1 depletion as both can be rescued by a Flag–HA-tagged HP1-rescue construct (Extended Data Fig. 4a). Data are mean ± s.d. Number of biological replicates: 6 for control; 7 for HP1-KD shRNA#1; shRNA#3 for HP1-KD shRNA#2. **e**, Box plot showing reduction of the HP1 ChIP–seq signal in HP1-KD embryos at zygotic genome activation on HP1 peaks at pericentromeric (PC) regions (left) and on HP1-peaks along the chromosome (Chr) arms (right). The signal is overall more reduced within pericentromeric regions compared to peaks along the chromosome arms. For comparison, quantitative ChIP–seq data using spike-in normalization have been used. See Supplementary Methods for definition of the pericentromeric regions. Box plots are as in Fig. 1d. **f**, Quantitative PCR (qPCR) measuring the upregulation of the telomeric repeat element Het-A caused by HP1-KD. The overexpression of Het-A can be rescued by the introduction of a HP1-rescue construct, that cannot be targeted by the hairpin. Data are mean ± s.d. Number of biological replicates: *n* = 4 for control; *n* = 4 for HP1-KD; 3 for HP1-rescue. **g**, Genome-wide Hi-C contact maps of control (left, 7 replicates) and HP1-KD (right, 5 replicates) embryos. **h**, Hi-C contact map in control (top) and HP1-KD (bottom) embryos across chromosome 2R 6–25 Mb at a resolution of 120 kb. Five biological replicates are shown. **i**, Hi-C contact enrichment in control (top) and HP1-KD (bottom) embryos, sorted by compartment score showing strong decrease in B-compartment contacts and gain in in A/B intermingling upon depletion of HP1. Quantification of the enrichment in compartment interactions is indicated in the respective corner of the plot. See Supplementary Methods for further details. **j**, Differential Hi-C contact enrichment in HP1-KD versus control Hi-C maps, sorted by compartment score for all individual replicates used in the study (top) and the individual chromosome arms (bottom), confirming the consistency of the phenotype across replicates and chromosome arms. **k**, Hi-C contact maps in control (left) and HP1-KD (right) embryos showing the inter-arm interactions (3L 2640000–14160000 and 3R 15840000–27240000) of chromosome 3 (left) as well as inter-chromosome interactions between chromosome 2L (6000000–17880000) and chromosome 3R (6600000-23760000). In both cases, contacts and compartmentalization are strongly reduced after HP1 knockdown. **l**, Scatter plot of compartment scores (first eigenvector values at 10-kb resolution) in control and HP1-KD embryos (Spearman correlation 0.85), indicating the complete absence of compartment switches between control and HP1-KD embryos. **m**, Hi-C contact map across a 1-Mb region on chr3L, showing decreased insulation across topologically associating domains in HP1-KD embryos. **n**, Insulation scores in ±100 kb surrounding TAD boundaries (Supplementary Methods) showing decreased insulation after HP1 depletion. **o**, Differential Hi-C contact enrichment in HP1-KD versus control Hi-C maps, sorted by compartment score for regions further apart than 500 kb (left) and regions further apart than 3 Mb (right). B-compartment contacts are also decreased at distances that exceed typical TAD sizes in *Drosophila*, which confirms that the moderately decreased insulation cannot account for the loss of B-compartment interactions. Source data

To explore the role of HP1 in establishing 3D chromosome organization, we examined early embryos that were depleted of maternally supplied HP1. Because HP1 is essential in *Drosophila*^15^, we performed conditional knockdown^22^ (Extended Data Fig. 3a, Supplementary Methods).

Complete depletion of HP1 blocked development before ZGA, whereas partial knockdown of HP1 still supported development to ZGA (Extended Data Fig. 3b, c, Supplementary Methods). Therefore, we used the partial HP1-knockdown (HP1-KD) embryos in all subsequent experiments. The embryonic lethality of the partial HP1-KD embryos was rescued with a short hairpin RNA (shRNA)-resistant HP1 (HP1-rescue) (Extended Data Fig. 3d), confirming the specificity. HP1 depletion led to strongly reduced binding of HP1 genome-wide, and to upregulation of the telomeric retroelement Het-A that was rescued in HP1-rescue embryos (Extended Data Figs. 1g, 3e, f).

**Fig. 3:**
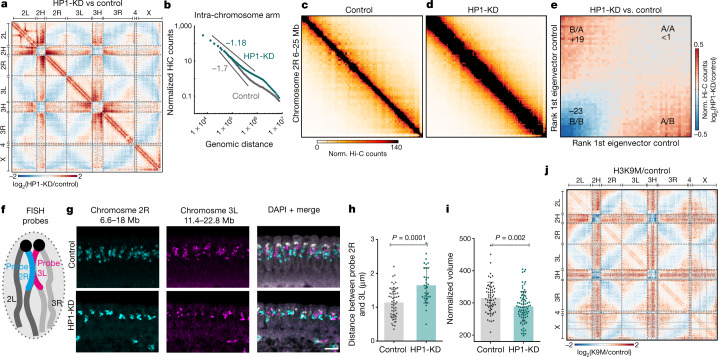
Depletion of HP1 causes increased intra-chromosome compaction and reduced compartmentalization. **a**, Differential Hi-C contact map (log_2_-transformed), highlighting increased contact frequencies within chromosome arms, decreased inter-arm and inter-chromosome contacts, reduced associations within and between pericentromeric regions, and increased interactions of pericentromeric regions with chromosome arms in HP1-KD embryos. Biological replicates were pooled; *n* = 7 control and *n* = 5 HP1-KD embryos. **b**, HP1-KD embryos show a milder decay of contact probabilities above 100 kb. **c**, Hi-C contact maps of 19 Mb on chromosome 2R in control embryos (resolution: 120 kb). **d**, As in **c**, in HP1-KD embryos. **e**, Differential contact enrichment in HP1-KD versus control embryos, sorted by compartment score (Supplementary Methods), shows decreased B-compartment interactions and increased A/B intermixing. Changes relative to the control. **f**, Scheme of FISH probe design to quantify inter-arm distance and intra-arm compaction. **g**, Representative 3D-DNA FISH staining of control and HP1-KD embryos at ZGA. Signals from probes on chromosome 2R and chromosome 3L are shown separately and merged with DAPI staining. Scale bar, 5 μm. **h**, Quantification of physical distances between FISH signals from chromosome 2R and 3L (mean ± s.d., nuclei: control *n* = 55, HP1-KD *n* = 35). **i**, Quantification of compaction of FISH signals from chromosome 2R (mean ± s.d., nuclei: control *n* = 63, HP1-KD *n* = 75). **j**, Differential Hi-C contact map (log_2_-transformed), highlighting decreased inter-arm and inter-chromosomal contacts, reduced associations within and between pericentromeric regions, and increased interactions of pericentromeric regions with chromosome arms in H3K9M embryos. Biological replicates were pooled; *n* = 7 control and *n* = 2 H3K9M embryos. See Supplementary Methods and Extended Data Fig. 5 for further details. *P* values were determined by Wilcoxon two-sided test. Source data

Hi-C analysis of HP1-KD embryos at ZGA revealed major genome-wide changes in chromosome organization (Fig. 3a, Extended Data Fig. 3g, h); we found perturbed Rabl configuration with decreased contact frequencies within and between pericentromeric regions and reduced inter-arm and inter-chromosomal contacts (Fig. 3a). Unexpectedly, we also observed increased intra-chromosomal contacts and milder decay of contact probabilities within chromosome arms (Fig. 3b–d), which suggests an overall increase in chromosome compaction within arms.

**Extended Data Fig. 4:**
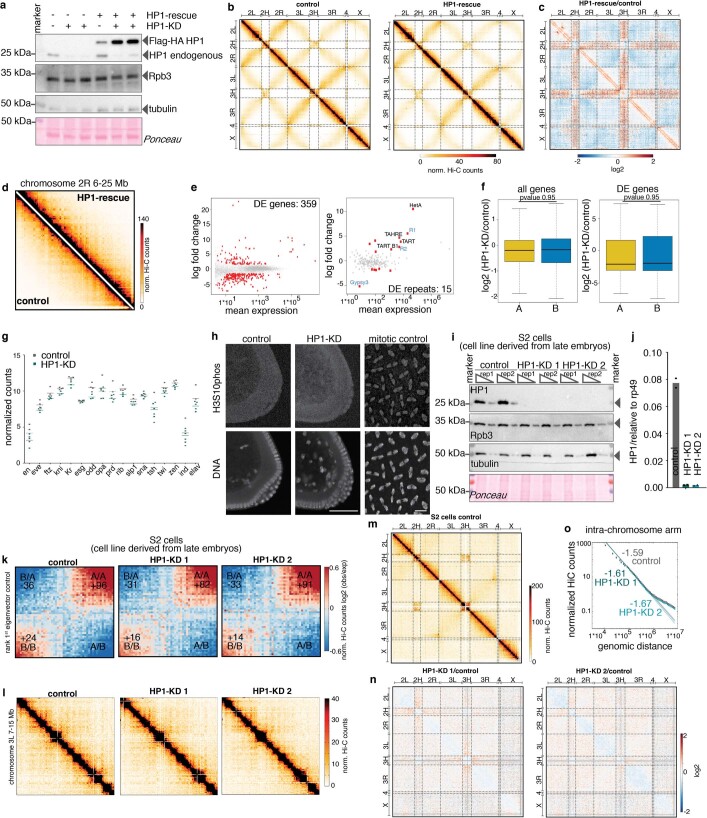
Characterization of HP1 rescue, transcriptomic changes after HP1 knockdown and its effect on 3D genome organization in differentiated S2 cells. **a**, Western blot showing the expression of the Flag-HA-tagged HP1 transgene in the background of control and HP1-KD embryos. Rpd3, tubulin and Ponceau were used as loading controls. After depletion of endogenous HP1, the expression of the transgene is increased. Blots are representative of two independent experiments. For western blot source data, see Supplementary Fig. 1. **b**, Genome-wide Hi-C contact maps in control (left) and HP1-rescue (right) embryos (40-kb resolution). The HP1-rescue and HP1-KD embryos show an inversion on chromosome 2L. **c**, Genome-wide differential Hi-C contact maps (log_2_-transformed fold change) in HP1-rescue versus control embryos. The HP1-rescue construct reverses the structural effects of HP1-KD (reduced contact frequency between the pericentromeric regions, as well as inter-chromosome arm interactions and compaction defects). **d**, Same genomic region as in Fig. 3c, d, with control and HP1-KD embryos expressing HP1-rescue. **e**, Left, MA plot illustrating differential expression of genes at zygotic genome activation in HP1-KD versus control embryos. In total, we detected 359 differentially expressed genes using RNA-seq (red dots) (Supplementary Methods) (of the total 277 genes are in A-compartment, 72 genes are in the B-compartment regions and 10 genes are on chrUn_CP007120v1). Right, MA plot showing the differential expression of types of repeat. We detected 15 differentially expressed repeat types, highlighted in the plot (Supplementary Methods). **f**, Box plot showing the distribution of gene expression changes within A- and B-compartments. We did not detect any differences in the distribution of gene expression changes in A- and B-compartments either considering all genes (left, *P* = 0.95, one-sided Wilcoxon test) or only significant differentially expressed genes (right, *P* = 0.95, one-sided Wilcoxon test). Box plots are as in Fig. 1d; outliers not shown. **g**, Expression of a panel of 17 purely zygotically expressed transcription factors in control and HP1-knockdown embryos. In unfertilized eggs all factors are not expressed and become upregulated at zygotic genome activation. The expression of the zygotic transcription factors confirms that HP1-KD embryos undergo zygotic genome activation. Each dot represents the normalized counts for a given transcription factor of a replicate RNA sequencing (RNA-seq) experiment. **h**, Immunofluorescence staining of control and HP1-KD embryos at zygotic genome activation with the mitosis marker H3S10 phosphorylated. Until the cellular blastoderm stage (ZGA), all nuclei undergo mitosis synchronously and then enter G2 phase at ZGA. The ratio of mitotic cells and the timing of mitosis is not altered in HP1-KD embryos. Scale bar, 50 μm. As a control for antibody specificity, an earlier stage of embryogenesis (before ZGA) was stained showing a strong H3S10phospho signal after synchronous entry into mitosis (right). Representative images from three biological replicates. Scale bar, 10 μm. **i**, Western blot showing the reduction of HP1 after treatment with double-stranded RNA (dsRNA) treatment in S2 cells (cell culture cells derived from a primary culture of late-stage (20–24 h old) *Drosophila* embryos, probably from a macrophage-like lineage). Rpd3, tubulin and Ponceau were used as loading controls. To control for unspecific effects of the dsRNA treatment, control cells were treated with a dsRNA against glutathione *S*-transferases (GST) and two different dsRNAs were used to deplete HP1. Representative of two independent experiments. For western blot source data, see Supplementary Fig. 1. See Supplementary Methods for further details. **j**, qPCR analysis showing the reduction of HP1 mRNA after dsRNA treatment in S2 cells. The signal is relative to *rp49*. To control for unspecific effects of the dsRNA treatment, control cells were treated with a dsRNA against GST and two different dsRNAs were used to deplete HP1. See Supplementary Methods for further details. Data are mean of two independent experiments. **k**, Hi-C contact enrichment in control (left) and HP1-KD (right) in S2 cells, sorted by compartment score, showing no decrease in B-compartment contacts after depletion of HP1 with either dsRNA. This indicates that HP1 is required for the establishment of the B-compartment during early embryonic development but does not affect the maintenance of compartmentalization in late differentiated cells. **l**, Hi-C contact frequencies of a 19-Mb region on chromosome 3L at a resolution of 120 kb. Pooled Hi-C data of two biological replicates are shown. **m**, Genome-wide Hi-C contact map in control S2 cells (120-kb resolution). **n**, Genome wide differential Hi-C contact maps (log_2_-transformed fold change) in HP1-KD versus control S2 cells. The differential contact maps show the HP1-KD with two independent shRNA on the left and right, respectively. **o**, Contact probabilities over the genomic distance of control and HP1-KD S2 cells. The contact probability of the HP1-KD cells closely resembles the control. Pericentromeric regions were excluded from the analysis of contact probabilities (Supplementary Methods). Source data

Notably, HP1-KD embryos also showed reduced segregation of A- and B-compartments, with a 20% decrease in B-compartment strength (Fig. 3e, Extended Data Fig. 3i, j). This effect was consistent across replicates, chromosome arms and for inter-arm and inter-chromosome contacts (Extended Data Fig. 3j, k). We found almost no compartment switching (Extended Data Fig. 3l). We also detected decreased insulation across topologically associating domains (TADs) (Extended Data Fig. 3m, n). By excluding short-range contacts (less than 500 kb or 3 Mb), we confirmed that the reduction of the B-compartment signal is independent of the reduction in TAD insulation (Extended Data Fig. 3o). Crucially, all of these phenotypes were rescued in HP1-rescue embryos (Extended Data Fig. 4a–d).

To validate the structural defects observed in HP1-KD embryos by Hi-C analysis, we performed 3D DNA fluorescence in situ hybridization (3D DNA FISH) with oligonucleotide probes spanning several megabases on chromosomes 2R and 3L (Fig. 3f, g). Quantitative image analysis of single cells showed that chromosomes were on average separated by larger distances (around 30% increase) in HP1-KD embryos (Fig. 3h, Supplementary Methods), in line with reduced inter-arm and inter-chromosome interactions observed in Hi-C data (Fig. 3a). In agreement with Hi-C data (Fig. 3b), we also found that the volume of the FISH signals was significantly decreased (around 10% decrease) (Supplementary Methods) in HP1-KD embryos (Fig. 3i), which suggests increased compaction of chromosome arms.

HP1 depletion thus perturbs the overall nuclear structure, with reduced proximity between pericentromeric regions, reduced alignment of chromosome arms and increased intra-chromosomal compaction. These global effects are accompanied by a prominent loss of contacts within B-compartment regions. The structural defects of HP1-KD embryos are notable, given that depletion of HP1 was only partial to allow embryos to reach ZGA. Our findings reveal that HP1 has a key role in establishing the 3D genome structure during development.

Only a small fraction of genes and repeats was misregulated in HP1-KD embryos at ZGA (Extended Data Fig. 4e). The most highly upregulated retroelements were localized at telomeric regions (Het-A, TAHRE and TART retrotransposons) and cannot account for the structural changes that we observed genome-wide (Extended Data Fig. 4e, f). We confirmed that HP1-KD embryos did not show defects in the onset of transcription at ZGA, and that both the control and the HP1-KD embryos at ZGA were in interphase (Extended Data Fig. 4g, h).

To investigate the role of HP1 in the establishment versus the maintenance of chromatin structures, we performed Hi-C experiments with differentiated, somatic *Drosophila* S2 cells. Notably, HP1 depletion did not considerably affect genome architecture (Extended Data Fig. 4i–o), which suggests that HP1 is not required to maintain chromatin structure.

**Extended Data Fig. 5:**
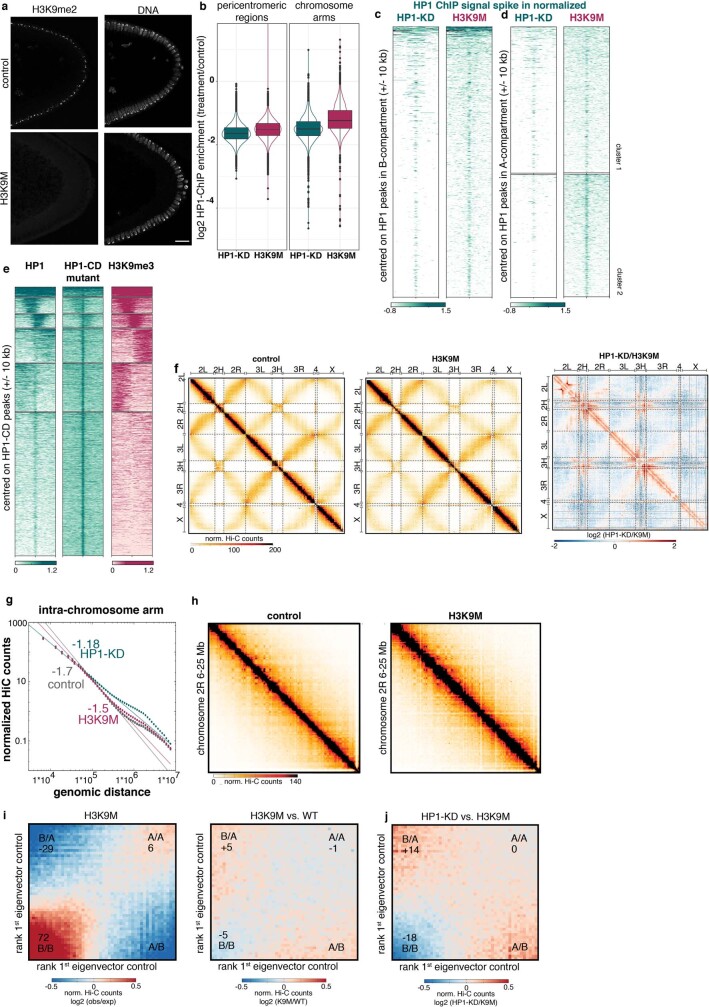
Characterization of H3K9M. **a**, Immunofluorescence staining of embryos at ZGA showing that H3K9me2 is completely lost after expression of the H3K9M mutant. H3K9M depletes H3K9me2/3 from chromatin, and acts as a competitive inhibitor of the histone methyltransferases. Representative image from three biological replicates. Scale bar, 20 μm. **b**, Box plot showing the reduction of the HP1 ChIP–seq signal over the control embryos at ZGA at HP1 peaks in HP1-KD (green) and H3K9M (red) embryos. The signal is reduced overall in HP1-KD embryos, with more loss in the pericentromeric region (left) compared to chromosome arms (right). For comparison, quantitative ChIP–seq data using spike-in normalization has been used. Box plots are as in Fig. 1d. **c**, Characterization of HP1 binding in B-compartment regions after HP1 knockdown (left) and H3K9M overexpression (right). Heat maps of HP1 ChIP–seq signals are ±10 kb centred on HP1 peaks and show that HP1 is retained at a higher level in H3K9M embryos compared to the HP1-KD embryos. Spike-in normalization has been used to quantify the enrichment. **d**, As in **c**, but in A-compartment regions. **e**, Characterization of HP1 binding in ovaries. Left, binding of a control HP1–Flag-HA-tagged transgene. Middle, binding of a Flag-HA-tagged chromodomain mutant of HP1 (HP1-CD) that cannot bind to H3K9me2/3. Right, the enrichment of H3K9me3 in ovaries. The heat maps of HP1 ChIP–seq signals are ± 10 kb centred on HP1 peaks called in the HP1 chromodomain mutant. **f**, Genome-wide Hi-C contact maps in control (left) and H3K9M (middle) embryos (120-kb resolution, pooled Hi-C data of two biological replicates). Right, differential Hi-C contact map (log_2_-transformed fold change in HP1-KD versus H3K9M), highlighting milder compaction within arms in H3K9M with respect to HP1-KD. **g**, H3K9M shows decay of contact probability similar to control embryos within arms. This suggests that compaction in the H3K9M mutant is milder than in HP1-KD embryos. **h**, Hi-C contact maps on chr2R (6–25 Mb) in control and H3K9M embryos (120-kb resolution). **i**, Hi-C contact enrichment in H3K9M (left) and differential Hi-C contact enrichment in H3K9M versus control (right) in embryos, sorted by compartment score showing no decrease in B-compartment contacts upon H3K9M expression. **j**, Differential Hi-C contact enrichment in HP1-KD versus H3K9M Hi-C maps, sorted by compartment score. B-compartment interactions are more strongly decreased in HP1-KD embryos than in H3K9M embryos. Source data

Because HP1 interacts with chromatin by binding to H3K9me2/3, we generated embryos depleted of H3K9me2/3 by overexpressing the histone 3 lysine 9-to-methionine (H3K9M) mutation^23^ (Extended Data Fig. 5a). Quantitative ChIP–seq for HP1 in precisely hand-staged H3K9M embryos at ZGA showed that HP1 binding was greatly reduced on pericentromeric and repeat regions as well as chromosome arms (Extended Data Fig. 5b–d). However, HP1 was 20% more retained on chromosome arms in H3K9M compared to HP1-KD embryos (Extended Data Fig. 5b, right), which could be due to some residual H3K9me2/3 and/or H3K9me2/3-independent binding of HP1 (Extended Data Fig. 5d, right, cluster 2). ChIP–seq analysis of chromodomain-mutant HP1 (HP1-CD)^13^ also revealed some residual binding on chromosome arms, further supporting H3K9me2/3-independent binding of HP1 (Extended Data Fig. 5e).

Hi-C maps of H3K9M embryos revealed pericentromeric heterochromatin de-clustering and reduced chromosome arm alignment, but only a mild gain in chromosome arm compaction and mild defects in compartmentalization (Fig. 3j, Extended Data Fig. 5f–j), which could be explained by higher retention of HP1 along chromosome arms in H3K9M embryos (Extended Data Fig. 5b).

Overall, our data indicate that HP1 has a major role in establishing chromatin architecture in early embryos by: (1) mediating the clustering and condensation of constitutive heterochromatin at pericentromeric regions through H3K9me2/3-dependent binding; (2) aiding the overall configuration of chromosome arms; and (3) contributing to the formation of the B-compartment.

**Fig. 4:**
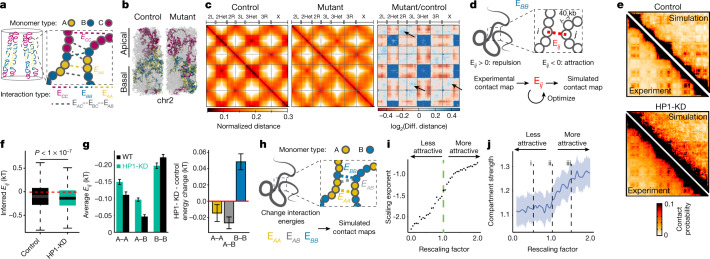
HP1 establishes de novo chromatin architecture during development via two independent mechanisms. **a**, Whole-genome polymer model. A- and B-type beads correspond to 10-kb A- and B-compartment regions. C-type beads correspond to pericentromeric and telomeric regions. **b**, Snapshots of wild-type control (left) and mutant (right) simulations. **c**, Genome-wide simulated distance maps of control (left) and mutant (centre). Right, differential distance map highlighting increased distances within and between centromeric and telomeric regions and reduced chromosome arm alignment (arrows). **d**, Polymer model of multi-megabase chromosome arm regions. Interaction energies between 40-kb beads are inferred to reproduce the experimental Hi-C map. **e**, Experimental and simulated contact maps in control (top) and HP1-KD (bottom) embryos (chr3R 17–20.6 Mb). **f**, Inferred interaction energies are overall more attractive in the HP1-KD model. *P* value determined by two-sided Wilcoxon test. Box plots are as in Fig. 1d. **g**, Left, interaction energies between B-type beads (B–B) become comparatively less attractive in HP1-KD embryos, but more attractive between A-type beads (A–A) and between A and B types (A–B). Right, average interaction energy changes between HP1-KD and control models. B-compartment attractions decrease in the HP1-KD model. Data are mean ± s.e.m., interactions: 990 (A–A), 2,069 (A–B), 1,035 (B–B). **h**, Chromatin is modelled as a chain of two types (A and B) of interacting 40-kb beads (chr3R 17–20.6 Mb). **i**, Scaling exponents increase when attractions between all beads are increased by a multiplicative factor, and vice versa. **j**, Compartment strength (bold line: mean) decreases when attractions between beads are increased, and vice versa. Confidence interval (shaded area) calculated using t-based approximation. Source data

**Extended Data Fig. 6:**
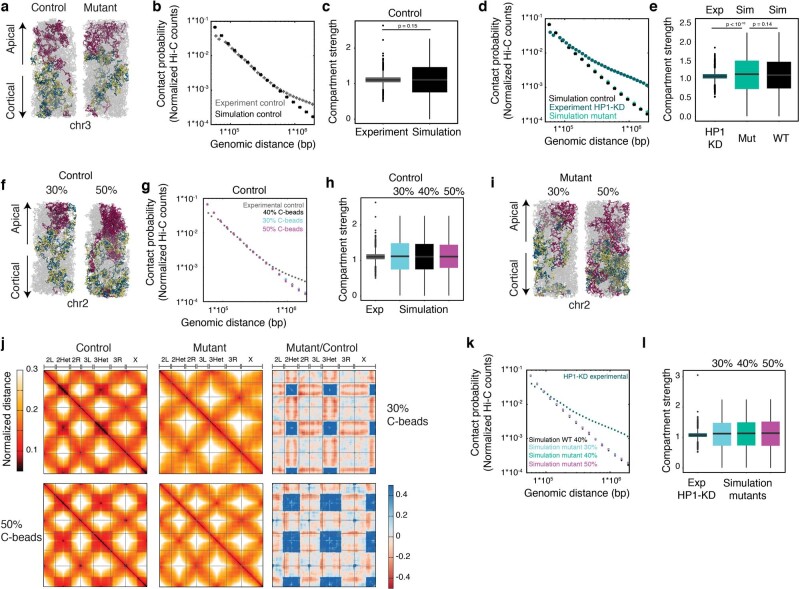
Genome-wide simulations show that loss of HP1 at pericentromeric regions does not cause the phenotype within chromosome arms. **a**, Snapshots of control (left) and mutant (decreased interactions between C-type beads and between C-type beads and the nuclear envelope, right) simulations, reproducing the experimental scaling and compartment strength. Colour code as in Fig. 4a. **b**, Scaling of contact probabilities in experimental and simulated contact maps. **c**, Simulated and experimental compartment strength. *P* values determined by two-sided Wilcoxon test. **d**, Scaling of contact probabilities in experimental HP1-KD and simulated control embryos and mutant contact maps. No differences between simulated control and mutant are detected. **e**, Experimental compartment strength in HP1-KD and simulated compartment strength in control and mutant samples. No differences between simulated control and mutant are detected. *P* values determined by two-sided Wilcoxon test. **f**, Snapshots of the control simulations with different amounts of C-type beads (30% and 50%). A single full chromosome is highlighted in each snapshot. **g**, Scaling of contact probabilities in experimental and simulated contact maps with different amounts of C-type beads. **h**, Experimental and simulated compartment strength with different amounts of C-type beads. **i**, Snapshots of the simulations with decreased interactions between C-type beads and their interaction with nuclear surface. A single chromosome is highlighted in each snapshot. Different amounts of C-type beads (30% and 50% of the total number of beads) are shown. **j**, Genome-wide simulated distance maps of control (left) and mutant samples with decreased interaction within and between C-type beads and their interaction with nuclear surface (middle). Differential distance map (log_2_-transformed fold change in mutant versus control) highlighting increased distance within and between centromeric and telomeric regions (right). Different amounts of C-type beads (30% and 50% of the total number of beads) are shown. **k**, Scaling of contact probabilities in experimental HP1-KD and simulated control and mutant contact maps with different amounts of C-type beads. No differences between simulated control and mutants are detected. **l**, Experimental compartment strength in HP1-KD and simulated compartment strength in mutants with different amounts of C-type beads. No differences between the simulated mutants are detected. Box plots are as in Fig. 1d. Source data

Next, we set out to exclude that folding defects observed at chromosome arms in HP1-KD embryos could arise as a mere consequence of the expansion of pericentromeric chromatin. Because it is impossible to completely decouple these effects in vivo, we turned to a genome-wide polymer modelling approach in which chromosomes are represented as chains of three types of 10-kb beads (A, B and C corresponding to A- and B-compartment and pericentromeric/telomeric regions, respectively) confined in a cylindrical nucleus (Fig. 4a, Supplementary Methods). We first optimized a set of interaction energies to reproduce contact probability scaling and compartment strength within arms in control embryos (Extended Data Fig. 6a–c). Next, we mimicked centromere de-clustering by decreasing interactions among C-type beads and their interactions with the nuclear surface (mutant) (Fig. 4b, c). The model recapitulated reduced alignment between chromosome arms (Fig. 4c, right) and increased interactions between pericentromeric regions and chromosome arms (Fig. 4c), but not compaction and compartmentalization defects within arms (Extended Data Fig. 6d, e). These results do not depend on the numbers of centromeric and telomeric beads (Extended Data Fig. 6f–l). This suggests that compartment defects and intra-arm compaction are a consequence of decreased HP1 binding on chromosome arms.

To understand the cause of compartment defects in HP1-KD embryos and determine whether they might simply arise from increased intra-arm compaction (Fig. 3a–d), we implemented two smaller-scale polymer models designed to uncover the energies driving the folding of chromosome arms.

**Extended Data Fig. 7:**
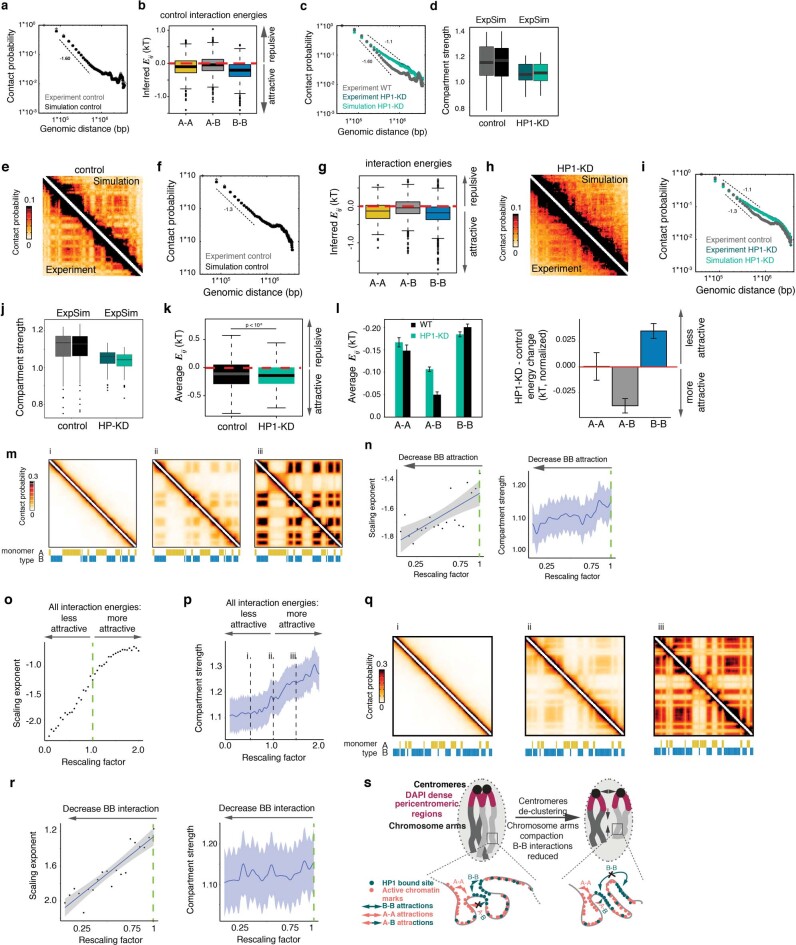
HP1-KD phenotype is driven by two independent mechanisms, both mediated by HP1. **a**, Scaling of contact probabilities in experimental and simulated contact maps shown in Fig. 4e. **b**, Inferred interaction energies between pairs of beads are overall attractive, with interactions between B-compartment beads being more attractive than interactions between A–A and A–B beads. Simulated region: chr3R 17–20.6 Mb. **c**, Scaling of contact probabilities in experimental and simulated Hi-C maps, compared to the scaling in the experimental control Hi-C heat map as a reference. Simulated region: chr3R 17–20.6 Mb. **d**, Simulated and experimental compartment strength (chr3R 17–20.6 Mb). **e**, Experimental and simulated contact maps of control embryos. Simulated region: chr3R 25.4–29 Mb. **f**, Scaling of contact probabilities in experimental and simulated contact maps shown in **e**. **g**, Inferred interaction energies between pairs of beads are overall attractive, with attractions between B-compartment beads being more attractive than interactions between A–A and A–B beads. Simulated region: chr3R 25.4–29 Mb. **h**, Experimental and simulated HP1-KD contact maps in the same region as in **e**. **i**, Scaling of contact probabilities in experimental and simulated Hi-C maps, compared to the scaling in the experimental control. Simulated region: chr3R 25.4–29 Mb. **j**, Simulated and experimental compartment strength in the same chr3R region. Simulated region: chr3R 25.4–29 Mb. **k**, Inferred interaction energies are overall more attractive in the HP1-KD model. *P* value determined by two-sided Wilcoxon test. Simulated region: chr3R 25.4–29 Mb. **l**, Left, interaction energies between B-compartment type beads become comparatively less attractive, whereas interactions between A-type compartment beads and between A- and B-type beads become more attractive. Data are mean and s.e.m. across each interaction energy class (number of interactions: 465 for A–A; 1,858 for A–B; and 1,769 for B–B). Right, average changes in inferred interaction energies between HP1-KD and the control models, classified according to whether they are within or across A- and B-compartment regions. Attractions between B-compartment regions are decreased in the HP1-KD model. Data are mean and s.e.m. across each interaction energy class (number of interactions: 465 for A–A; 1,858 for A–B; and 1,769 for B–B). Average changes in inferred interaction energies between HP1-KD and the control models, irrespective of being within or across A- and B-compartment regions, are set to zero. Interaction energies between B-compartment type beads become less attractive, whereas energies between A-type compartment beads and between A and B become more attractive. **m**, Example of the simulated contact maps for different levels of compartment strength, corresponding to different energy rescaling factors (i, ii and iii, as in Fig. 4j). Arrangement of A and B beads based on: chr3R 17–20.6 Mb. **n**, Same plot as in Fig. 4i, j, when only attractions between B-compartment regions are decreased. Mean (bold line) with the confidence interval (shaded area) calculated using t-based approximation is shown. Arrangement of A and B beads based on: chr3R 17–20.6 Mb. **o**, Scaling exponents in simulated contact maps are plotted against increasing or decreasing (by a multiplicative scaling factor) A–A, A–B and B–B attractive interaction. The scaling exponent increases when increasing attractions between all beads and vice versa. Arrangement of A and B beads based on chr3R 25.4–29 Mb. **p**, Compartment strength in simulated contact maps decreases upon increase in attractions between all types of bead, and vice versa. Mean (bold line) with the confidence interval (shaded area) calculated using t-based approximation is shown. Arrangement of A and B beads based on chr3R 25.4–29 Mb. **q**, Example of the simulated contact maps for different levels of compartment strength, corresponding to different energy rescaling factors. Arrangement of A and B beads based on chr3R 25.4–29 Mb. **r**, As in **o** and **p**, when only attractions between B-compartment regions are decreased. Mean (bold line) with the confidence interval (shaded area) calculated using t-based approximation is shown. Arrangement of A and B beads based on chr3R 25.4–29 Mb. **s**, Proposed model in which chromatin-bound HP1 mediates B–B attractions. A–A attractions independent of HP1 promote establishment of the A-compartment. Depletion of HP1 causes pericentromeric region declustering and increased chromosome arm compaction. B–B interactions are reduced, leading to an overall increase in A–A and A–B attractive energies (Supplementary Methods, Extended Data Fig. 6). Box plots are as in Fig. 1d; outliers not shown in **k**. Source data

In the first approach, interaction energies between 40-kb beads were optimized to reproduce experimental Hi-C maps within multi-megabase regions of chromosome arms^24,25^ (Fig. 4d, Supplementary Methods). For control contact maps (Fig. 4e, top), we found that interaction energies were globally attractive, which accounts for the correct contact probability scaling (Extended Data Fig. 7a), The model predicted that A–A and B–B interactions were on average more attractive than A–B interactions (Extended Data Fig. 7b). For HP1-KD contact maps (Fig. 4e, bottom, Extended Data Fig. 7c, d), we found increased attractions overall between all bead types but comparatively less attractive B–B interactions (Fig. 4f, g). Notably, our findings do not depend on the specific region that is simulated (Extended Data Fig. 7e–l). This suggests that decreased compartmentalization is not a mere consequence of increased compaction after HP1 knockdown (Fig. 3a–d) but instead requires the simultaneous loss of B-specific attractive interactions.

To confirm these findings, we used a more general model that is not designed to reproduce the experimental Hi-C maps but instead describes the behaviour of a polymer when interaction energies between its constituent A- and B-type beads are systematically varied (Fig. 4h, Supplementary Methods). Increasing all A–A, A–B and B–B interaction energies correctly predicted milder scaling of contact probabilities (such as HP1-KD), but led to stronger compartments (Fig. 4i, j, Extended Data Fig. 7m). By contrast, decreasing all interaction energies correctly predicted compartment loss but led to the wrong scaling behaviour (steeper decay) (Fig. 4i, j, Extended Data Fig. 7m). Finally, decreasing only B–B attractions reproduced the observed decrease in compartment strength but resulted in a steeper scaling (Extended Data Fig. 7n). Thus, modifying chromosome compaction alone cannot explain the HP1-KD structural phenotype, which suggests that HP1 depletion perturbs compartmental forces. Notably, these results do not depend on the distribution of A- and B-compartment beads (Extended Data Fig. 7o–r). Analysis of this general polymer model shows that the HP1-KD structural phenotype within arms (increased compaction, lower compartmentalization) arises from two independent mechanisms: decreased specific interactions between B-compartment regions, and increased attraction between all genomic locations.

Our data and modelling approaches suggest that HP1-mediated interactions, which might occur through HP1 oligomerization^14^ or phase separation^20,21^, have a major role in establishing 3D genome conformation during embryogenesis. Decreased HP1 binding in pericentromeric heterochromatin led to declustering and decondensation of constitutive heterochromatin and a perturbed Rabl configuration. By contrast, decreased HP1 levels within chromosome arms caused decreased B–B compartment attractions and increased arm compaction, possibly owing to decreased chromatin stiffness. Reduced segregation of B-compartment regions after HP1 knockdown might facilitate interactions between A- and B-type chromatin and allow attractions between active regions to dominate, resulting in globally increased compaction (Extended Data Fig. 7s). This is consistent with quantitative compartment analysis (Fig. 3e, Extended Data Fig. 3i, j) and the overall increase in A–A and A–B interactions in simulations (Fig. 4g). Alternatively, increased attractions could arise from HP1 counteracting condensin II-mediated homologous chromosome pairing or cohesin-mediated loop extrusion.

In the A-compartment, HP1-mediated compartmental forces might be counteracted by surrounding active chromatin modifications such as H3K9ac (Fig. 2d, Extended Data Fig. 7 s). Because the A-compartment is not affected after disruption of the B-compartment (Fig. 3e), we suggest that it is controlled by a distinct driving force independent of HP1.

Our study shows that HP1 is required to establish pericentromeric heterochromatin clustering in early embryos but is dispensable in differentiated cells, consistent with a recent report in mammals^26^. In differentiated cells, clustering might be driven by other HP1 paralogues or heterochromatin proteins^2^ favoured by the slower cell cycle, or result from other mechanisms involving solid-like states in heterochromatin condensates^27^. We also showed that HP1 prevents the collapse of chromosome arms while they elongate to establish the characteristic Rabl configuration. Finally, HP1 is directly involved in the formation of the B- but not the A-compartment region. Because pericentromeric clustering and compartmentalization also occur in mammals, HP1 could have similar functions during mammalian embryogenesis.

### Reporting summary

Further information on research design is available in the Nature Research Reporting Summary linked to this paper.

## Online content

Any methods, additional references, Nature Research reporting summaries, source data, extended data, supplementary information, acknowledgements, peer review information; details of author contributions and competing interests; and statements of data and code availability are available at 10.1038/s41586-021-03460-z.

## Supplementary information

Supplementary Information This file contains all Materials and Methods as well as Supplementary Fig.1 with all raw images of the Western Blots performed in the study.

Reporting Summary

Supplementary Table 1 Compartments called using HiTC in control embryos at ZGA.

Supplementary Table 2 Compartments called using HiTC in HP1-KD embryos at ZGA.

Supplementary Table 3 Drosophila genomic regions annotated as pericentromeric heterochromatin.

Supplementary Table 4 UCSC annotation of Drosophila repeats.

Supplementary Table 5 HP1 peaks called with MACS2 using the broad peaks option before cycle 9.

Supplementary Table 6 HP1 peaks called with MACS2 using the broad peaks option between cycle 9-13.

Supplementary Table 7 HP1 peaks called with MACS2 using the broad peaks option at ZGA.

Supplementary Table 8 RNA-Seq count table comparing the HP1-KD and control embryos at ZGA.

## Data Availability

All Hi-C, ChIP–seq and RNA sequencing raw files generated in this study have been uploaded to the Gene Expression Omnnibus (GEO) under accession GSE140542. The following public databases were used: BSgenome.Dmelanogaster.UCSC.dm6, org.Dm.eg.db and TxDb.Dmelanogaster.UCSC.dm6.ensGene. Source data are provided with this paper.

## Source data

Source Data Fig. 1 Source Data Fig. 3 Source Data Fig. 4 Source Data Extended Data Fig. 1 Source Data Extended Data Fig. 2 Source Data Extended Data Fig. 3 Source Data Extended Data Fig. 4 Source Data Extended Data Fig. 5 Source Data Extended Data Fig. 6 Source Data Extended Data Fig. 7

## Code availability

Custom code generated in this study is available at: https://github.com/zhanyinx/Zenk_Zhan_et_al_Nature2021.
