# Supplementary material for: HP1 drives de novo 3D genome reorganization in early Drosophila embryos
Source: Nature. 2021 Apr 14;593(7858):289–93. doi: 10.1038/s41586-021-03460-z (PMC8116211; doi:10.1038/s41586-021-03460-z)

nature portfolio

https://doi.org/10.1038/s41586-021-03460-z

Supplementary information

HP1 drives de novo 3D genome reorganization in early *Drosophila* embryos

In the format provided by the
authors and unedited

Supplementary Information HP1 drives de novo 3D genome reorganization in early Drosophila embryos

Supplementary Methods

RNAi meditated knockdown of target genes

In order to induce RNAi mediated silencing of certain gene products, we employed the Gal4-UAS system. By crossing males of a Gal4 driver line (Bloomington #7063) to females of the fly RNAi collection TRiP[28] we obtained an F1 generation depleted for HP1 in the female germ line (Bloomington #33400). These females were crossed to siblings and produced embryos that were depleted of HP1 mRNA and protein (Extended Data Fig. 3a). The flies were reared under standard conditions and transferred to cages to collect the embryos for further experiments. The same driver line and strategy was used to overexpress transgenes in the female germ line and early embryos.

Embryo collection

To collect precisely time staged embryos the flies were allowed to lay eggs on apple juice agar plates in cages for one hour at 25 °C. In order to synchronize the laying of the flies, plates were changed for three consecutive times, before the collection and the embryos were discarded. After three pre-lays, the embryos were collected for one hour and aged for another two hours at 25 °C to reach the stage of zygotic genome activation. To enrich earlier developmental stages, embryos were collected for 30 min on apple juice agar plates and aged at 25 °C according to the desired stage (before c9 for 30 min and cycle 9-13 for 70 min).
The plate with the embryos was removed from the incubator and the embryos were dechorionated with 50% bleach, washed with water and carefully dried. The embryos were transferred in 7 ml of heptane and 5 ml of freshly prepared fixative were added. The fixative consists of buffer A (60 mM KCl, 15 mM NaCl, 15 mM HEPES [pH 7.6], 4 mM $MgCl_2$) and 1% of formaldehyde for ChIP or 1.8% of formaldehyde for HiC. We also used DSG (disuccinimidyl glutarate) for fixation in order to crosslink loosely bound complexes to chromatin and investigate the localization of HP1 more in detail[29]. For DSG fixation the embryos were first incubated for 45 min in heptane and a fixative (buffer A with 2 mM DSG) followed by a second formaldehyde fixation for 15 min.
During the formaldehyde fixation the embryos were incubated on an orbital shaker at maximum speed for 15 min. The fixation was stopped by the addition of 225 mM glycine (final concentration). The embryos were incubated for 5 min on a wheel and washed with buffer A + 0.1% Triton. After washing, the embryos were hand-sorted on a cooling station under a microscope with transmitted light.
During early development, all nuclei divide in the syncytial blastoderm. The number of cycles corresponds to the mitotic divisions. At cycle 9 the nuclei migrate to the periphery of the embryo and an initial subset of 100 genes is transcribed. The migration of the nuclei to the periphery of the embryo can be used as readout to follow the early developmental cycles (see Fig. 1a).
The typical clear rim and the cellular membranes that form at zygotic genome activation characterizes this developmental stage. At stage 5 (ZGA), the cell cycle slows down and the embryos synchronously enter and pause in G2 phase (that lasts around 30 min)[30].
The hand-sorted embryos, at ZGA, were shock frozen and stored in batches of 400 for ChIP experiments and in batches of 50-100 for HiC experiments at -80 °C.

Cloning of transgenes and production of transgenic flies

The transgenes for HP1-rescue and H3K9M overexpression were amplified from cDNA of 0-3 hr embryos. Restriction sites and a Flag-HA tag were added to the N-terminal part of the HP1-rescue construct and the C-terminal end of H3K9M through the primer. The mutations of Tryptophan 45 to Alanine and Tyrosine 48 to Alanine in the chromo domain of HP1 were added by fusion PCR with two primers flanking the mutations and the outer primers, that were used to clone HP1[31]. Lysine 9 of H3.3 was mutated to methionine by quick exchange. Both transgenes were cloned into pUASp-attB (DGRC_1358) and integrated into the locus 22A3 (fly line: y[1] w[1118]; PBac(y[+]-attP-3B)VK00037). A stronger short hairpin RNA against the 3'UTR of HP1 was generated following the guidelines of the TRIP collection[28,32] and cloned in the vector VALIUM20.

Primers used for cloning:

FZ262_SpeI_FH_HP1_fw	actagtATGGATTACAAGGATGACGATGACAAGCTCGATGGAGGATACCCCTACGACGTG CCCGACTACGCCGGAGGACTCGAGGGCAAGAAAATCGACAACCCTGAG
FZ263_HP1_BamHI_rev	ggatccTTAATCTTCATTATCAGAGTACCAGG
FZ324_HP1_W45A_Y48A_fw	gcGAAGGGCgcTCCCGAAACTGAGAACACG
FZ325_HP1_W45A_Y48A_rev	gcGCCCTTCgcTTTCAGATAGTACTCCACC
VA01_SpeI_H3.3_fw	actagtTATGGCACGTACCAAGCAAACAGCC
FZ83_H3.3A_FLAG-HA_rev	CTAGGCGTAGTCGGGCACGTCGTAGGGGTATCCTCCAGCGGCCGACTTGTCATCGTCAT CCTTGTAATCTCCTCCAGCGGCCGCGGCCCGCTCGCCACGGATGCG
VA02_HA_BamHI_rev	ggatccCTAGGCGTAGTCGGGCACGTCG
quick exchange K9M fw	AGCCCGTATGTCGACCGGA
quick exchange K9M rev	TCCGGTCGACATACGGGCT
FZ266_bottomstrand_oligo_HP1_3UTR_1	aattcgcAGAACGATTATACATTTAACGtatgcttgaatataactaCGTTAAATGTATAATCGTTCTactg
FZ267_topstrand_oligo_HP1_3UTR_1	ctagcagtAGAACGATTATACATTTAACGtagttatattcaagcataCGTTAAATGTATAATCGTTCTgcg

HP1 knockdown in S2 cells

In order to deplete HP1 mRNA in S2 cells, oligos were designed (http://www.dkfz.de/signaling/e-rnai3/) that allowed the production of a T7 binding site coupled 150 bp long PCR products including the coding sequence of third or the fourth exon of the HP1 transcript. Two independent regions were chosen to control for off-target effects. The regions were amplified from cDNA of 0-3 hr embryos. As a control a 300 bp region of GST was amplified from the vector pET41a. The PCR product was gel-purified and 500 ng -1 µg were used for *in-vitro* transcription using the HiScribe T7 In Vitro Transcription Kit (NEB E2050) running the reaction overnight. The fragments were purified using the MEGAclear transcription clean-up kit (Invitrogen AM1908). The RNA was eluted twice with EB-buffer heated to 95 °C. The RNA was aliquoted and stored at -80 °C.

To treat S2 cells with the dsRNA, cells were seeded in Express Five SFM (Gibco 10486025) supplemented with GlutaMAX (Gibco 35050061) at 1 Mio cells/ml in 6 ml shaking flasks. The dsRNA was thawed on ice and heated to 95 °C for 5 min, then the heating block was switched off and the dsRNA was allowed to cool down to 40 °C before it was transferred to a metal rack at RT for 10 min and then transferred to ice. The dsRNA was diluted in 250 µl of Express Five SFM to reach a final amount of 60 µg of dsRNA per sample (10 µg of dsRNA in 1 Mio cells). To increase transfection efficiency 15 µl of Lipofectamine RNAiMAX (Invitrogen 13778100) were also diluted in 250 µl of Express Five SFM. The Lipofectamine mix and the diluted dsRNA were mixed and incubated for 15 min at RT. The Lipofectamine-dsRNA mix was added to the cells. The cells were grown shaking for 2 days, when a boost of dsRNA (5 µg/1 Mio cells) was added. After 4 days the cells had reached a density of 5-6 Mio cells/ml and knockdown efficiency was assessed by Western Blot and qPCR. To perform HiC the cells were harvested by centrifugation at 1300 g for 5 min, washed with buffer A (supplemented with 10 mM of sodiumbutyrate) and fixed for 10 min at RT by adding 1 ml of 1% PFA diluted in buffer A (supplemented with 10 mM of sodiumbutyrate). The fixation was stopped by adding 100 µl of 2.5 M glycine and the cells were immediately pelleted 1300 g for 5 min. The cells were washed again twice with buffer A (supplemented with 10 mM of sodiumbutyrate), shock frozen and stored until further use at -80 °C.

Primers used for PCR product generation:

FZ463_HP1-KD_dsRNA_1_fw	GtaatacgactcactatagggACAGATGCGGAGCAGGACACC
FZ464_HP1-KD_dsRNA_1_rev	GtaatacgactcactatagggGAGGGCACCATTTCTGCTTGG
FZ465_HP1-KD_dsRNA_2_fw	GtaatacgactcactatagggAAGTCAGCCGCCTCCAAGAAGG
FZ466_HP1-KD_dsRNA_2_rev	GtaatacgactcactatagggGTTGGTTCTTCGGACTTTCGC
GST_dsRNA_fw	TtaatacgactcactatagggAGATATCAATTTGTGGGATAGCT
GST_dsRNA_rev	TtaatacgactcactatagggAGATTTTGGATATTAGATACGGT

HiC

Embryos were defrosted in ice-cold 50 µl HiC lysis buffer (10 mM Tris-HCl [pH 8], 10 mM NaCl, 0.2% Igepal, 1x Protease Inhibitor Cocktail (Roche, 11836170001)) on ice and immediately crashed with a pestle for 30 sec. The crashing of the embryos was checked under a microscope. For S2 cells the nuclei were released using the NEXSON protocol[33]. Briefly, the cells were resuspended in 1 ml of ice-cold

HiC lysis buffer and subjected to ultrasound in a Covaris sonicator (using the following parameters peak power 75, duty factor 2, cycles/burst 200, 67 sec). The integrity of the nuclei was checked under the microscope.
The nuclei were pelleted for 5 min at 3000 g (rcf) and 4 °C. After discarding the supernatant, the pellet was incubated in 50 µl 0.5% SDS for 10 min at RT. The reaction was quenched by adding 145 µl of water and 25 µl of 10% Triton X100.
To digest the chromatin 25 µl of DpnII buffer were added and 0.7 µl of DpnII (NEB R0543T). The reaction was incubated for 90 min at 37 °C in a thermomixer at 600 rpm and a second instance of 0.7 µl of DpnII was added followed by a second incubation of 90 min.
The nuclei were pelleted for 5 min at 1000 g (rcf) at RT and resuspended in 135 µl of 1x CutSmart buffer (NEB B7204S). To biotinylate the free chromatin ends, dNTP-mix (NEB) (final 0.15 mM), Biotin-14-dATP (Jena Bioscience, NU-835-BIO14-S) (final 0.05 mM) and 10 U of Klenow (NEB M0210 L) were added to the reaction (final volume 150 µl) and incubated for 60 min at 25 °C. Next the free ends of the chromatin were ligated in a total volume of 1200 µl by adding ligation buffer (NEB B0202S) (1x final), 10% Triton (0.8% final), 120 µg BSA, 2000 U ligase (NEB M0202S). After two hours a second instance of 2000 U ligase was added and incubated for another 2 hours. Following the incubation, the nuclei were pelleted at 1000 g for 5 min at RT, the supernatant was discarded and the nuclei resuspended in 200 µl elution buffer (10 mM Tris-HCl [pH 8], 1 mM EDTA, 1% SDS). Proteins were digested upon addition of 400 µg Proteinase K for 30 min at 55 °C. The chromatin was reverse crosslinked in the presence of 0.365 M NaCl at 68 °C overnight.
The next day DNA was purified using the ChIP DNA Clean&Concentrator Kit (Zymo Research D5205) eluted in 50 µl and biotin was removed from unligated fragments by adding NEB buffer 2 (NEB B7002S) (1x final), 12 µg of BSA, dATP and dGTP (0.025 final each), 3 U T4 DNA polymerase (NEB M0203S) in a final volume of 120 µl. The reaction was stopped by adding EDTA to a final concentration of 13 mM.
The DNA was transferred to Covaris microTUBE snap cap (520045) and sheared using the Covaris E220 (with intensifier (50014) and the following parameters duty factor 10%, peak incident power 140, cycle/burst 200, 120 sec. The DNA fragments were in the range of 200-300 bp.
Ligated and biotinylated fragments were enriched through biotin pulldown using Dynabeads MyOne Streptavidin C1 (Invitrogen 65001). The samples were filled up to a total of 400 µl with 2x binding buffer (10 mM Tris-HCl [pH 8], 1 mM EDTA, 2 M NaCl) (1x final) and beads (equilibrated with TWB (5 mM Tris-HCl [pH 8], 0.5 mM EDTA, 1 M NaCl, 0.05% Tween) resuspended in 1x binding buffer were added. The beads were washed twice for 2 min at 55 °C with TWB and twice with EB-buffer (Qiagen). Last the beads were resuspended in 50 µl of EB-buffer and directly used for library preparation using the NEB Ultra II DNA Library Prep Kit for Illumina (E7645S and E6440 or E7335, E7500, E7710, E7730) following the manufacturer's instructions except that the adapter ligation happened on the biotin beads. The fragments were released from the beads before the PCR reaction (by heating to 98 °C for 10 min) after unligated adapters had been removed. To amplify the libraries 12-13 cycles of PCR were performed.

ChIP
All ChIP experiments were performed as described earlier[22,34]. With the change that libraries were prepared using NEB Ultra II DNA Library Prep Kit for Illumina (E7645S and E6440 or E7335, E7500, E7710, E7730). All ChIPs were performed during embryonic development and the stages are indicated in the figure descriptions. Except the ChIP experiment to map the binding of the HP1-chromodomain mutant. Given the embryonic lethality of this mutation, we performed HP1 ChIP-seq in the adult germline in order to map H3K9me2/3 independent binding of HP1. This confirms, that HP1 can bind independently of H3K9me2/3 to non-repetitive regions enriched in active histone marks
All antibodies used for ChIP were validated also by western blot immunofluorescence or ChIP in knockdown embryos of the respective protein, methyl- or acetyltransferase. For HP1 we generated binding profiles using antibodies against the endogenous protein and another antibody against the tagged transgene and obtained similar results. For HP1 also a different crosslinking strategy using DSG (disuccinimidyl glutarate, a longer crosslinker) was applied in order to also study loose interactions of HP1.
In order to quantitatively assess the reduction of HP1 binding in the background of different mutants (HP1-KD and K9M) lambda DNA spike ins were added during the process. To perform a quantitative

ChIP experiment all samples were processed in parallel starting from the same amount of embryos. After the preclearing a 20 µl aliquot of the sample was reverse crosslinked overnight at 65 °C and treated with RNAse and Proteinase K. The subsample was used to calculate the total amount of chromatin in the sample. All samples were then adjusted to contain the same amount of chromatin. Of the chromatin solution 10% were kept as an Input and lambda DNA was added at a final dilution of 1:1000 (usually around 60 pg of lambda DNA, then IPs were performed from 600 ng of total chromatin).
To account for differences in the amount of immunoprecipitated DNA 2 pg of lambda DNA per sample were added to the elution buffer. The lambda DNA was diluted in elution buffer and the same batch of buffer was used to elute all samples processed in parallel.
All antibodies used in this study can be found in the Western Blot section.

Immunofluorescence and Imaging

Embryos were collected on apple juice agar plates for 0-4 hrs. The embryos were dechorionated using 50% bleach and transferred in to 1:1 heptane:fixative (4% formaldehyde in 1xPBS) solution. The embryos were incubated for 20 min on an orbital shaker. To devitillinize the embryos, the aqueous phase was removed and methanol was added followed by vortexing. Devitillinized embryos sink to the bottom of the tube and can be used for immunofluorescence stainings.
For stainings the embryos were washed with 1xPBS+0.2% Triton and blocked with 1% BSA before they were incubated for 2 h at room temperature or overnight at 4 °C with the primary antibody. After washing with 1xPBS+0.2% Triton, the secondary antibody and DAPI were incubated for another 2 h at room temperature. VectaShield (Vector Laboratories H-1000) was used as mounting medium.
All images were acquired at the confocal laser scanning microscope Zeiss ELYRA PS1. For high resolution microscopy Zeiss Airyscan was used. Stacks were assembled using Fiji[35] or Imaris 9.5.1 (Bitplane). All antibodies used in this study can be found in the Western Blot section.

Oligopaint in *Drosophila* embryos

Embryos were collected on apple juice agar plates for 0-4 hrs and dechorionated as described above. The embryos were fixed for 20 min on an orbital shaker in a 1:1 mixture of heptane and fixative (4% formaldehyde in buffer A FISH (60 mM KCl, 15 mM NaCl, 15 mM Pipes [pH 7,4], 2 mM EDTA [pH 8], 0.5 mM EGTA [pH 8], 0.5 mM Spermidine, 0.15 M Spermine)). The embryos were then devitillinized as described above and stored in methanol at -20 °C until further use. The staining was mostly performed as described in[36] with small modifications for oligopaint probes. To rehydrate the embryos and prepare them for staining they were incubated in a serial dilution of methanol in 1xPBS+0.1% Tween ranging from 90%, 70%, 50%, 30% down to 0% of methanol. Following the embryos were treated overnight with 200 µg/ml of RNAseA in 1xPBS+0.1% Tween at 4 °C on a rotating wheel.
The next day the embryos were permeabilized for 3 hrs at RT in 1xPBS+0.3%Triton on the nutator. The embryos were then passed through a serial dilution of pHM with 1xPBS+0.3%Triton (pHM pre-Hybridization Mixture: 50% deionized formamide, 4xSSC, 100 mM NaH_2PO_4 [pH 7], 0.1% Tween) to dilute out the 1xPBS+0.3%Triton. The steps of the dilution were 20% pHM, 50% pHM, 80% pHM and two washes at 100% pHM. Subsequently the DNA was denatured by heating the embryos to 82 °C for 15 min in 500 µl of pHM in a thermomixer. The first 10 min of the incubation the embryos were gently agitated, then they were allowed to settle and the supernatant was removed leaving around 30-50 µl of pHM behind. Of the probes 60 pmol were diluted in 30 µl of FHB (FISH Hybridization buffer: 2xSSC, 10% Dextran Sulfate, 50% deionized formamide, 0.05% Salmon sperm). The probe was denatured for 5 min at 80 °C and immediately added to the embryos (still incubating at 82 °C). Following the reaction was strictly protected from light and transferred to 37 °C and incubated overnight shaking at 900 rpm.
The next day the embryos were washed for 20 min at each step in 1 ml of the following posthybridization solutions.

1. 50% formamide, 2xSSC, 0.3% CHAPS shaking (900 rpm) at 37 °C
2. 40% formamide, 2xSSC, 0.3% CHAPS shaking (900 rpm) at 37 °C
3. 30% formamide, 1xPBS+0.1% Tween shaking (900 rpm) at 37 °C
4. 20% formamide, 1xPBS+0.1% Tween shaking (900 rpm) at 37 °C
5. 10% formamide, 1xPBS+0.1% Tween on a nutator at RT
6. 1xPBS+0.1% Tween, 10 µg DAPI on a nutator at RT
7. 1xPBS+0.1% Tween, 10 µg DAPI on a nutator at RT

After finishing the washes, the embryos were washed again twice with 1xPBS+0.1% Tween before they were mounted in Vectashield.

The probes used in this study were labelled with either Atto550 or Atto488 synthesized by BioCat GmbH in Heidelberg. A schematic is shown in Figure 3i. They cover the following regions:

2R_cen	chr2R	Chromosome 2R 6,000,000-18,013,000	Atto550
3L_cen	chr3L	Chromosome 3L 11,457,000-22,870,000	Atto488

Cellular Fractionation

Embryos were collected for 0-4 hrs or overnight on apple juice agar plates. After dechorionation the embryos were snap-frozen in liquid nitrogen and stored at -80 °C until further processing. To obtain the extracts the embryos were crashed with 10-12 strokes with a douncer tissue homogenizer in hypotonic buffer (15 mM HEPES [pH 8], 350 mM sucrose, 5 mM $MgCl_2$, 10 mM KCl, 0.1 mM EDTA, 0.5 mM EGTA, 10 mM beta-Mercaptoethanol, 0.2 mM PMSF) and incubated for 15 min on ice. The nuclei were pelleted at 4 °C and 9,000 g for 15 min and the nuclear soluble fraction was extracted for 15 min on ice by the addition of 1.5-10 pellet volumes of 1:3 high salt buffer (20 mM HEPES [pH 7.9], 25% glycerol, 1.5 mM $MgCl_2$, 1.2 M KCl, 0.2 mM EDTA, 0.2 mM PMSF, 0.5 mM DTT) and low salt buffer (20 mM HEPES [pH 7.9], 25% glycerol, 1.5 mM $MgCl_2$, 20 mM KCl, 0.2 mM EDTA, 0.2 mM PMSF, 0.5 mM DTT). Following a centrifugation at 4 °C, 20,000 g for 15 min the chromatin fraction was collected as the insoluble pellet that remained after the high salt extraction. The fractions were quantified by Bradford and equal amounts were analyzed by western blot.

Embryo collection for RNA

Embryos were collected on apple juice agar plates. In order to hand-stage, the embryos they were submerged with halocarbon oil 27 (Sigma Aldrich H8773) to allow hand-staging under a stereoscope with transmitted light. Embryos of the correct developmental stage were then transferred individually into 50 µl of Trizol (ThermoScientific, 15596026), crashed with a tissue grinder, snap-frozen and stored at -80 °C until RNA extraction.

RNA extraction and qPCR

The Trizol solution was defrosted on ice filled up to 500 µl and incubated for 5 min at room temperature. After the addition of 100 µl chloroform and vigorous mixing, the samples were spun in a table top centrifuge at 4 °C, 12,000 g for 15 min in order to separate the organic and aqueous phase. The aqueous phase was transferred in to 300 µl of chloroform and the step the first step was repeated. Subsequently the aqueous phase was transferred into isopropanol containing 20 µg of glycogen. The RNA was precipitated at -20 °C at least overnight and pelleted by centrifugation (4 °C, 12.000 g for 60 min). The pellet was washed with 80% ethanol and resuspended in DNase-digestion buffer (2 U TurboDNase Ambion, 1x TurboDNase buffer Ambion). After digestion for 30 min at 37 °C the reaction was stopped by adding EDTA to a final concentration of 15 mM and heating the samples to 75 °C for 10 min. The RNA was quantified using Qubit RNA BR Assay kit (ThermoScientific Q10210) and integrity was assessed on agarose gels or the fragmentanalyzer (Agilent).

The Illumina TruSeq Stranded Total RNA protocol was used for library preparation for next generation sequencing.

To prepare cDNA for qPCR 150 ng of total RNA were used following the manufacturer's instruction (RevertAid First Strand cDNA Synthesis Kit, ThermoScientific, K1621).

To measure mRNA expression by qPCR the cDNA was diluted 1:10 in water and added to a mastermix containing the primers and FastStart Universal SYBR Green Master (Rox) (Sigma Aldrich, 4913850001) and the LightCycler 96 (Roche) (qPCR program: Preincubation: 600 sec at 95 °C, 2 Step Amplification: 15 sec at 95 °C, 60 sec at 60 °C – repeat 55x, Melting Curve: 15 sec at 95 °C, 60 sec at 60 °C, 1 sec at 95 °C, Cooling: 30 sec at 37 °C). The expression of the target was normalized to rp49.

The primers used for qPCR are:

o767_Het_fw	CGCAAAGACATCTGGAGGACTACC
o768_Het_rev	TGCCGACCTGCTTGGTATTG
o111_ 5'RT-RP49_pair2	CTAAGCTGTCGCACAAATGG
o112_3'RT-RP49_pair2	GGGCATCAGATACTGTCCCT
o677_HP1-su(var)205_qPCR_2fw	AGTACGCCGTGGAAAAGATCA

o678_HP1-su(var)205_qPCR_2rev CGTGTTCTCAGTTTCGGGATAG

Phenotypical characterization of developmental phenotypes

To determine the hatching rate of embryos, the embryos were collected for 0-1 h on apple juice agar plates. From the plates 120 embryos were randomly picked per experiment and transferred to a new plate in groups of 10. The hatching rate was calculated per group of 120 embryos and was plotted in a graph. For cellularization rate, 50 embryos were transferred to a new plate and monitored until they reached zygotic genome activation, which can be easily recognized by the specific morphology of the embryo. The cellularization rate was calculated per group of 50 embryos and was plotted in a graph.

Western Blot

Embryos were collected into Laemmli-Buffer (2 embryos per µL), crashed with a pestle, subsequently run on SDS-PAGE (BioRad System) and transferred to PVDF membrane using Wet-Blot. The ECL signal was recorded using Amersham Hyperfilm ECL (GE Healthcare Life Sciences) or ChemiDoc Imaging System (Bio-Rad). Loading controls were probed on the same membrane and loading was also controlled by Ponceau staining. All raw data are provided in Supplementary Fig.1.

The antibodies used in this study

antibody	Immunofluorescence	ChIP	western		origin/manufacturer
HP1	1:20	1-2 µg	1:1000	mouse	Developmental Studies Hybridoma Bank C1A9
HP1	-	1 µg	-	rabbit	Covance, PRB-291C-200
HA (C29F4)	1:100	5 µl	-	rabbit	Cell Signaling #3724S
HA.11	1:100	-	1:5000	mouse	Covance MMS-101P
H3K9ac	-	1 µg	1:5000	rabbit	ActiveMotif 39586
H3K9ac	-	1 µg	1:5000	rabbit	ActiveMotif 39918
H3K9me3	-	1 µg	-	rabbit	ActiveMotif 39161
H3K9me2	1:100	1 µg	-	rabbit	ActiveMotif 39239
H3K9me3	1:100	-	-	rabbit	Abcam ab176916
H3K27me3		1 µg		rabbit	Diagenode, C15410195 (Lot A1811-001P)
H3K4me1		1 µg		rabbit	Diagenode, C15410194 (Lot A1862D)
H3K4me3		1 µg		rabbit	Diagenode, C15410003
H3K27ac		1 µg		rabbit	Diagenode, C15410196 (Lot A1723-041D)
Rpb3 (PolII)		3 µl		rabbit	Carla Margulies Lab
Tubulin DM1	-	-	1:10000	mouse	Sigma T9026
H3	-	-	1:10000	mouse	ActiveMotif MABI 0301
Rpb3 (PolII)	-		1:5000	rabbit	Asifa Akhtar Lab

Hi-C analysis

Hi-C data were analysed using HiC-Pro version 2.11.1[37] with *--very-sensitive --end-to-end --reorder* option (full parameters at https://github.com/zhanyinx/Zenk_Zhan_et_al_Nature2021). Briefly, read pairs were mapped to the *D. melanogaster* (build dm6), recovering chimeric reads after recognition of the ligation site. Only unique valid pairs were used to build contact maps at different binning sizes after dividing the genome into equally sized bins. Iterative correction (ICE)[38] was then applied on binned data. In the genome-wide maps, 2x2 pixel averaging was performed for clearer visualization of the contact map.

Compartments analysis

To call compartments at high resolution, we followed the strategy described in[39] (code at https://github.com/zhanyinx/Zenk_Zhan_et_al_Nature2021). We used Hi-C data at 10 kb, and divided each chromosome arm into 2.56 Mb regions. We then called compartments on the corresponding 2.56 Mb Hi-C matrix using HiTC[40]. The identity of A and B compartments in S2 cells is based on gene density. In embryo, we used the number of H3K27ac peaks called using the IDR (irreproducible discovery rate) filter. Briefly, H3K27ac ChIP-seq reads were mapped using bowtie with -n 2 -l 28 -e 70 -k 1 -m 10 -X 500 parameters. Then peaks were called for each replicate using macs2 with -p 0.01. IDR scores were calculated using IDR software, and peaks are filtered by imposing IDR >= 0.01. The compartment scores are provided as Supplementary Tables (1-Zenk-Zhan-Supplementary_Tables-compartments_control.txt, 2-Zenk-Zhan-Supplementary_Tables_compartments_HP1-KD.txt)

To create the contact enrichment plots (saddle plots), we first called compartments chromosome-arm wide using HiTC on Hi-C at 10kb resolution after removing the pericentromeric regions.

We defined the following regions as pericentromeric (the genomic coordinates of pericentromeric regions are also provided as Supplementary Tables (3-Zenk-Zhan-Supplementary_Tables_pericentromeric_regions.txt)).

Chromosome 2L	22000000-23513712
Chromosome 2R	1-6000000
Chromosome 3L	23000000-28110227
Chromosome 3R	1-4100000
Chromosome X	21500000-23542271

We then sorted the distance normalized interaction matrix of each chromosome arm based on the first eigenvector. To reduce the noise, we kept only interactions between monomers below 5 Mb and filtered out the top 0.1 percentile interactions. We then re-binned the resulting matrix into a 50x50 matrix and averaged across arms. We then re-binned the resulting matrix into a 50x50 matrix and averaged across arms to create saddle plots. To allow comparison between conditions and across organisms, saddle plot is then normalized to the control, producing differential saddle plots. Change in compartmentalization is finally quantified by calculating the mean value of the 10 bins in each corner of the differential saddle plot. In particular, we used the geometric mean of interactions between 10 bins containing the strongest B compartments (bottom left corner in the saddle plot) as enrichment of interaction between B compartment regions. For A compartments, we used 10 bins corresponding to the strongest A (top right corner in the saddle plot). The full custom script can be found at https://github.com/zhanyinx/Zenk_Zhan_et_al_Nature2021.

Compartments strength

To calculate the compartment strength, we used the same definition as in[1]:

$$Score(b_{i\epsilon A}) = \frac{< C_{ij} >_{j\epsilon A}}{< C_{ij} >_j}$$

$$Score(b_{i\epsilon B}) = \frac{< C_{ij} >_{j\epsilon B}}{< C_{ij} >_j}$$

Where b_i is the genomic bin *i*, C_{ij} is the distance normalized Hi-C interaction in cis between bin *i* and *j* and $<>_j$ denotes the average over j

Insulation score:

To calculate the insulation score around TAD boundaries, we called TADs using 6.4 kb binned Hi-C data in control. We used the directionality index software[41] using the following parameters: window=100000, min=1 and prob=0.99.

Insulation scores were calculated using the matrix2insulation.pl from[42] with the following parameters: -is 64000 -ids 6400 -im mean -bmoe 3 -nt 0.1.

Scaling analysis:

To calculate the scaling of contact probabilities from Hi-C data, we used logarithmic binning with a binning size of 0.05 in log10. Pericentromeric regions were excluded from this analysis. To compare the scaling of experimental data and the corresponding simulation, we used linear binning with bin sizes equal to the Hi-C resolution. Hi-C data binned at 6.4 kb were used to create scaling plots in Fig. 3b, at 10 kb for the genome-wide modelling (Fig. 4) and at 40kb for the smaller scale modelling (Fig. 4).

ChIP sequencing analysis:

Reads were mapped to the *D. melanogaster* genome (build dm6) using qAlign from QuasR package[43] using the following alignments parameters: -n 2 -l 28 -e 70 -k 1 -X 500. This allows to keep reads mapping to multiple loci and randomly assigns them to one of the multiple locations. In case of Lambda DNA spike-in, the same parameters were used to map the unmapped reads to Lambda genome. ChIP enrichment over input was calculated using bamCompare from deepTools[44] using the following parameters: --scaleFactorsMethod SES --smoothLength 900 --binSize 300. In case of spike-in normalization, the relative Dm6/lambda total number of reads for both input and IP was provided to

bamCompare for normalisation through the option --scaleFactors. Peaks were called using macs2[45], using a cut-off of 0.05 on the q-value. --broad option was added for the H3K9me3 and HP1 ChIP.
The coverage heatmaps around peaks were generated using plotHeatmap from deepTools[44]. For Figure 1c, samples were sorted according to the mean enrichment of HP1 at cycle 9-13. For Figure 2c, samples were sorted according to the mean enrichment of HP1 at zygotic genome activation. For Figure 2d, k-mean clustering was applied with 2 clusters and samples were sorted according to the mean enrichment of all samples.
The location of HP1 peaks and position of repeat elements is provided as Supplementary Tables (4-Zenk-Zhan-Supplementary_Tables_drosophila_repeats_UCSC_annotation.txt and 5-Zenk-Zhan-Supplementary_Tables_HP1_broad_peaks_bc9.txt, 6-Zenk-Zhan-Supplementary_Tables_HP1_broad_peaks_c-9-13.txt, 7-Zenk-Zhan-Supplementary_Tables_HP1_broad_peaks_ZGA.txt

Quantification of relative HP1 ChIP enrichment in totipotent and stage 5 embryo:
To quantify the relative enrichment in HP1 binding, we used spike-in corrected enrichment. We quantified median enrichment of HP1 signal under the peaks detected at stage 5. Since the bulk of HP1 dissociates from chromatin during mitosis[46] and early embryos undergo very rapid and synchronous mitotic divisions, we corrected the enrichment in totipotent nuclei (before cycle 9) for the presence of mitotic nuclei, in order to compare the amount of HP1 on chromatin before and at stage 5. We estimated that 50% of the nuclei at the totipotent stage (before cycle 9) are mitotic[47-49]. By correcting for mitotic nuclei, we estimated that the enrichment of HP1 binding in totipotent nuclei (before cycle 9) is ~16% of the enrichment at stage 5.

RNA sequencing analysis
Reads were mapped to the *D. melanogaster* genome (build dm6) using STAR[50], using the following options: --outSJfilterReads Unique --outFilterType BySJout --outFilterMultimapNmax 1000000 --alignSJoverhangMin 6 --alignSJDBoverhangMin 2 --outFilterMismatchNoverLmax 0.04 --alignIntronMin 20 --alignIntronMax 1000000 --outSAMstrandField intronMotif --outFilterIntronMotifs RemoveNoncanonicalUnannotated --seedSearchStartLmax 50 --twopassMode basic. Gene expression was quantified through qCount from QuasR package[43] using the "TxDb.Dmelanogaster.UCSC.dm6.ensGene" database for gene annotation and UCSC repeatMasker table for repeats annotation. The following repeat families were excluded from the analysis: simple repeats, low complexity, artefact, satellite, other and unknown.
To call differentially expressed genes and repeats, we used DESeq2[51] and defined differentially expressed genes using the following thresholds: absolute log-foldchange higher than log2(3) and q-value smaller than 0.01. A count table comparing the HP1-KD and control embryos is provided as Supplementary Tables (8-Zenk-Zhan-Supplementary_Tables_RNA_seq_count_table.xls).

Image analysis to quantify distance, compaction, and measure HP1 distribution
Distance between center of mass and volume were quantified using custom macro routines in Fiji. Briefly, we crop the region of interest (ROI) of size 60x60x7 pixels centered around the FISH signal. If the ROI goes outside the image, then the image border is used to crop the ROI. We transform the cropped image into binary mask using Fiji default method for center of mass and max entropy method for volume estimation. The distance between centers of mass is the Euclidian distance between the centers of mass. The effective volume is estimated as the average number of non-zero pixels across z-stacks in the binary mask. The custom scripts can be found at https://github.com/zhanyinx/Zenk_Zhan_et_al_Nature2021.
To quantify the relative amount of HP1 co-localizing with H3K9me3 in the pericentromeric regions shown in Extended Data Figure 1b, we used Imaris 9.5.1 to define surface area for the HP1 and H3K9me3 immunofluorescence signal using the default parameters. We then quantified the average signal intensity of the HP1 (normalized by area size) within the HP1 surface (mean intensity=1.26) and the H3K9me3 surface (mean intensity=33.06). We quantified the average signal in about 300 nuclei from 9 different individual embryos. We used the same images to measure the aspect ratio of a nucleus at stage 5, used for the genome wide simulations. We find that on average a nucleus at stage 5 is around 10x5x5 µm.

Multi-Mb scale simulations:
To simulate the chromatin fiber, we employed coarse-grained polymer models that describe each 40-kb region as a monomer. In the first approach (Fig. 4d-g, Extended Data 7a-l), beads were not *a priori* assigned to either A- or B-compartments. Instead, their mutual interaction energies were learned directly from the Hi-C data in an unsupervised iterative manner so that the resulting equilibrium ensembles of fiber conformations had the same contact frequencies as those observed in the Hi-C experiment (Simulated region in Fig. 4e-g and Extended Data Fig. 7a-d: chr3R 17-20.6 Mb; in Extended Data Fig. 7e-l: chr3R 25.4-29 Mb). This is done applying the principle of maximum entropy[52], which in the present context can be implemented specifying a contact potential
$U = \sum_{i<j}\left[B_{HC}\Theta\left(R_{HC} - |r_i - r_j|\right) + B_{ij}\Theta(R - |r_i - r_j|)\right]$,
where $\Theta(r)$ is a step function which takes the value 1, if its argument is positive and 0 if it is negative, R_{HC} is a hard-core radius, R is the interaction range, B_{ij} (expressed in kT, where k is the Boltzmann constant and T is the temperature) is the interaction energy of the specific pair of monomers, and B_{HC} is the hard-core energy, that we set to $+\infty$.
Assuming that the Hi-C map reports equilibrium contact probabilities between chromosome regions, one has to find the matrix of energies B_{ij} for which the contact probabilities f_{ij} obtained from the model are equal to the experimental ones f_{ij}^{exp}. This is done through an iterative Monte Carlo scheme[24] which samples the conformations of the polymer at equilibrium and adjusts the values of B_{ij} to minimize the $\chi^2 = 2N^{-1}(N-1)^{-1}\sum_{i<j}\left(f_{ij} - f_{ij}^{exp}\right)^2/\sigma_{ij}^2$, where σ_{ij} are the experimental standard deviations obtained from triplicate experiments.
We used the MonteGrappa code version 1.2[53] to perform the simulations. For a given genomic region, the optimal potentials were inferred using the parameters at https://github.com/zhanyinx/Zenk_Zhan_et_al_Nature2021. All the optimal potentials can also be found at https://github.com/zhanyinx/Zenk_Zhan_et_al_Nature2021.
The second modelling approach (Fig. 4h-j, Extended Data Fig. 7m-r), is not designed to reproduce the experimental Hi-C maps but rather aims to study the behaviour of a polymer when interaction energies between its constituent beads are systematically varied. In this modelling approach, each bead was assigned *a priori* to either the A- or B-compartments based on compartments called on experimental control maps at 40 kb resolution (chromosome 3R 17-20.6 Mb for Fig. 4h-j, Extended Data Fig. 7m,n and chromosome 3R 25.4-29 Mb for Extended Data Fig. 7o-r). A- and B-type beads interacted with three types of interaction energies (A-A, A-B and B-B) when they are sufficiently close in space. The reference interaction energies (rescaling equal to 1) between the two types of monomers are set to the average optimal interaction energies inferred to reproduce the control Hi-C heatmap in the same region. We carried out 120 independent simulations and used all the recorded conformations to calculate the scaling of contact probability and the compartment strength.

Genome-wide simulations
To perform molecular dynamics (MD) simulations of the whole diploid genome, we used the LAMMPS engine[54]. Chromosomes were modelled as bead-and-string polymers at the resolution of 10 kbp and were constrained in a cylinder of radius 24.84 a.u. and height 99.39 a.u. that mimics the nuclear envelope (aspect ratio derived from DAPI quantification, see Extended Data Fig. 1b). The pairs of chromosomes 2 and 3 started from a Rabl-configuration; chromosome 4 and mitochondrial chromosomes were not modelled because of their small size.
The monomers mimicking the telomeric region (3-Zenk-Zhan-Supplementary_Tables_pericentromeric_regions.txt) are tethered to the lower surface of the cylinder by attaching a single monomer to the lower surface, while the monomers mimicking the centromeric region (3-Zenk-Zhan-Supplementary_Tables_pericentromeric_regions.txt) are tethered to the upper surface by attaching a single monomer to the upper surface. For the X chromosome, we tethered only ends to the top and to the bottom of the cylinder. The number density of the polymer within the cylinder is 0.2.
Monomers were classified into three types: monomers correspond to the active (type A), to inactive chromatin (type B) within arms and pericentromeric/telomeric monomers (type C). Type-C monomers correspond to the 40% of the total genome (90% for pericentromeric and 10% for telomeric regions). Active and inactive chromatin monomers were assigned according to compartments called using Hi-C contact matrix at 10kb resolution.

Consecutive monomers of the polymer interact with the harmonic potential
$U_h = \frac{k}{2}\sum_i\left(|r_i - r_j| - a\right)^2$,
where k=40 (in arbitrary units) and a=1. All masses are set to 1. These quantities define the elementary scales of the system. The Boltzmann constant is set to 1, so temperatures are expressed in energy units below.
Monomers interact via the Lennard-Jones potential
$U = \sum_{i<j} 4\varepsilon_{ij}\left[\left(\frac{\sigma}{|r_i-r_j|}\right)^{12} - \left(\frac{\sigma}{|r_i-r_j|}\right)^{6}\right]$,
that defines the interaction between the three types of monomers and with the lamina in the wild-type system. To simulate the control condition, we tuned the interaction parameters to reproduce the scaling of contact probability and compartmental strength. This led to the following 3x4 matrix for ε_{ij}:

	A	B	C
A	0.25	0.15	0.15
B	0.15	0.315	0.2
C	0.15	0.2	0.33
Lamina	0.15	0.15	0.33

To mimic the de-clustering and detachment of centromeric and telomeric regions from the nuclear surface, starting from the control interaction energies, we set the C-C and C-Surface to 0.02.

	A	B	C
A	0.25	0.15	0.15
B	0.15	0.315	0.2
C	0.15	0.2	0.02
Lamina	0.15	0.15	0.02

The position of the well is at σ=1.2246. A cut-off is applied at $r_c = 2.5$.
We performed all the simulations in NVT ensemble. Initial conformations are obtained from a random walk constrained in a V-shaped tube (straight for X chromosomes). The equations of motions are integrated with velocity-Verlet algorithm with a time step of 0.001. To be sure that the results of the simulation did not depend on the initial condition, each simulation was carried out as follows: 1) the initial conformation was equilibrated by the Polak-Ribiere version of the conjugate gradient algorithm for up to 10^4 steps. 2) We generated random velocities for T=1.0 and run a MD simulation of the system for 10^7 steps, setting ε_{ij}=0.15 for all monomers and increasing smoothly the temperature to T=3.0. 3) We equilibrated the system at T=3.0 for 10^8 steps. 4) Using the monomer-dependent interactions ε_{ij}, we annealed smoothly the system to the temperature to T=1.0 for 10^6 steps. 5) We collected single conformation at T=1.0. For each set of parameters, we performed 50 independent runs and analyzed the overall set of collected conformations.
Distance maps display the average distances between monomers, considering both the two copies of experimentally indistinguishable chromosomes. To calculate the scaling of contact probability, we used a contact radius of 1.5.

Normalisation of Hi-C counts and inference of contact probabilities
Given that chromatin has a persistence length of around 3kb[55], we assumed that adjacent monomers of 3kb are always in contact and that

$$C_{ij} \approx \frac{1}{|i-j|^b}$$

which in log scale becomes

$$\log(C_{ij}) = a + b * |i-j|$$

where C_{ij} represents the Hi-C counts between genomic region i and j, b is the scaling exponent.
By estimating a and b using Hi-C data at 40kb, we can predict the C_{ij} at $|i-j| = 3kb$. Since in 40kb there are 40/3 monomers of 3kb, the normalization constant will be $C_{|i-j|=3k}/(\frac{40}{3} * (\frac{40}{3} - 1))$ where $(\frac{40}{3} * (\frac{40}{3} - 1)$represents the number of combinations of pairs of 3kb monomers in a 40kb monomer.

Statistical tests

We used the Wilcoxon one-sided two samples method to test the differential gene expression distribution between A and B compartment. We used the Wilcoxon two-sided two samples method to test the distribution of inferred energies between control and HP1-KD.

Data availability

All Hi-C, ChIP and RNA-Seq raw files generated in this study has been uploaded to GEO (GSE140542, https://www.ncbi.nlm.nih.gov/geo/query/acc.cgi?acc=GSE140542). Public databases used: BSgenome.Dmelanogaster.UCSC.dm6, org.Dm.eg.db, TxDb.Dmelanogaster.UCSC.dm6.ensGene.

Code availability

All custom codes used in the paper can be found on Github at https://github.com/zhanyinx/Zenk_Zhan_et_al_Nature2021

28 Ni, J. Q. *et al.* A genome-scale shRNA resource for transgenic RNAi in Drosophila. *Nature methods* **8**, 405-407, doi:10.1038/nmeth.1592 (2011).

29 Nowak, D. E., Tian, B. & Brasier, A. R. Two-step cross-linking method for identification of NF-kappaB gene network by chromatin immunoprecipitation. *Biotechniques* **39**, 715-725, doi:10.2144/000112014 (2005).

30 Edgar, B. A. & O'Farrell, P. H. The three postblastoderm cell cycles of Drosophila embryogenesis are regulated in G2 by string. *Cell* **62**, 469-480, doi:10.1016/0092-8674(90)90012-4 (1990).

31 Ostapcuk, V. *et al.* Activity-dependent neuroprotective protein recruits HP1 and CHD4 to control lineage-specifying genes. *Nature* **557**, 739-743, doi:10.1038/s41586-018-0153-8 (2018).

32 Perkins, L. A. *et al.* The Transgenic RNAi Project at Harvard Medical School: Resources and Validation. *Genetics* **201**, 843-852, doi:10.1534/genetics.115.180208 (2015).

33 Arrigoni, L. *et al.* Standardizing chromatin research: a simple and universal method for ChIP-seq. *Nucleic acids research* **44**, e67, doi:10.1093/nar/gkv1495 (2016).

34 Iovino, N., Ciabrelli, F. & Cavalli, G. PRC2 controls *Drosophila* oocyte cell fate by repressing cell cycle genes. *Dev Cell* **26**, 431-439, doi:10.1016/j.devcel.2013.06.021
S1534-5807(13)00384-5 [pii] (2013).

35 Schindelin, J. *et al.* Fiji: an open-source platform for biological-image analysis. *Nature methods* **9**, 676-682, doi:10.1038/nmeth.2019 (2012).

36 Bantignies, F. & Cavalli, G. Topological organization of Drosophila Hox genes using DNA fluorescent in situ hybridization. *Methods Mol Biol* **1196**, 103-120, doi:10.1007/978-1-4939-1242-1_7 (2014).

37 Servant, N. *et al.* HiC-Pro: an optimized and flexible pipeline for Hi-C data processing. *Genome Biol* **16**, 259, doi:10.1186/s13059-015-0831-x (2015).

38 Imakaev, M. *et al.* Iterative correction of Hi-C data reveals hallmarks of chromosome organization. *Nature methods* **9**, 999-1003, doi:10.1038/nmeth.2148 (2012).

39 Wang, Y. *et al.* Reprogramming of Meiotic Chromatin Architecture during Spermatogenesis. *Mol Cell* **73**, 547-561 e546, doi:10.1016/j.molcel.2018.11.019 (2019).

40 Servant, N. *et al.* HiTC: exploration of high-throughput 'C' experiments. *Bioinformatics* **28**, 2843-2844, doi:10.1093/bioinformatics/bts521 (2012).

41 Dixon, J. R. *et al.* Topological domains in mammalian genomes identified by analysis of chromatin interactions. *Nature* **485**, 376-380, doi:10.1038/nature11082 (2012).

42 Crane, E. *et al.* Condensin-driven remodelling of X chromosome topology during dosage compensation. *Nature* **523**, 240-244, doi:10.1038/nature14450 (2015).

43 Gaidatzis, D., Lerch, A., Hahne, F. & Stadler, M. B. QuasR: quantification and annotation of short reads in R. *Bioinformatics* **31**, 1130-1132, doi:10.1093/bioinformatics/btu781 (2015).

44 Ramirez, F. *et al.* deepTools2: a next generation web server for deep-sequencing data analysis. *Nucleic acids research* **44**, W160-165, doi:10.1093/nar/gkw257 (2016).

45 Zhang, Y. *et al.* Model-based analysis of ChIP-Seq (MACS). *Genome Biol* **9**, R137, doi:10.1186/gb-2008-9-9-r137 (2008).

46 Fischle, W. *et al.* Regulation of HP1-chromatin binding by histone H3 methylation and phosphorylation. *Nature* **438**, 1116-1122, doi:10.1038/nature04219 (2005).

47 Hartenstein, V. *Atlas of Drosophila development*. Vol. 328 (Cold Spring Harbor Laboratory Press, 1993).

48 Shermoen, A. W., McCleland, M. L. & O'Farrell, P. H. Developmental control of late replication and S phase length. *Current biology : CB* **20**, 2067-2077, doi:10.1016/j.cub.2010.10.021 (2010).
49 Blumenthal, A. B., Kriegstein, H. J. & Hogness, D. S. The units of DNA replication in Drosophila melanogaster chromosomes. *Cold Spring Harb Symp Quant Biol* **38**, 205-223, doi:10.1101/sqb.1974.038.01.024 (1974).
50 Dobin, A. *et al.* STAR: ultrafast universal RNA-seq aligner. *Bioinformatics* **29**, 15-21, doi:10.1093/bioinformatics/bts635 (2013).
51 Love, M. I., Huber, W. & Anders, S. Moderated estimation of fold change and dispersion for RNA-seq data with DESeq2. *Genome Biol* **15**, 550, doi:10.1186/s13059-014-0550-8 (2014).
52 Tiana, G. & Giorgetti, L. Integrating experiment, theory and simulation to determine the structure and dynamics of mammalian chromosomes. *Curr Opin Struct Biol* **49**, 11-17, doi:10.1016/j.sbi.2017.10.016 (2018).
53 Tiana, G. *et al.* MonteGrappa: An iterative Monte Carlo program to optimize biomolecular potentials in simplified models. *Computer Physics Communications* **186**, 93-104 (2015).
54 Plimpton, S. Fast parallel algorithms for short-range molecular dynamics. (Sandia National Labs., Albuquerque, NM (United States), 1993).
55 Redolfi, J. *et al.* DamC reveals principles of chromatin folding in vivo without crosslinking and ligation. *Nat Struct Mol Biol* **26**, 471-480, doi:10.1038/s41594-019-0231-0 (2019).

Supplementary Figure 1: Raw scans of all western blots presented in the study. The corresponding Extended Data Figure is indicated on top. The red area marks the parts shown in the Extended Data.

Extended Data Fig 1c

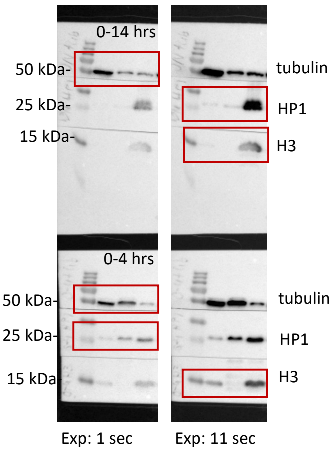

Extended Data Fig 3b

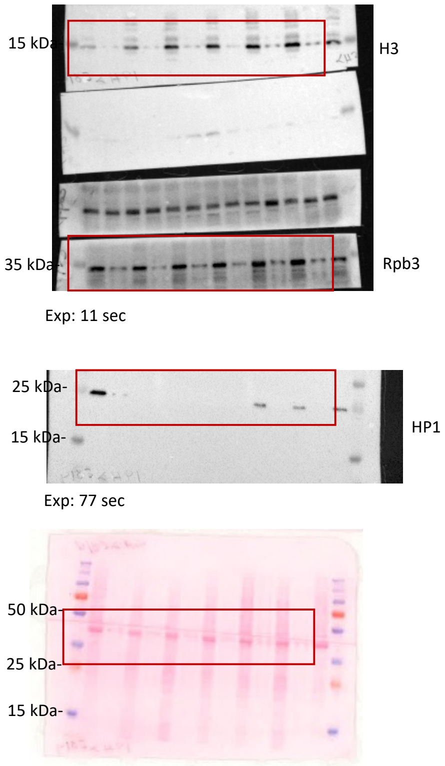

Extended Data Fig 4a

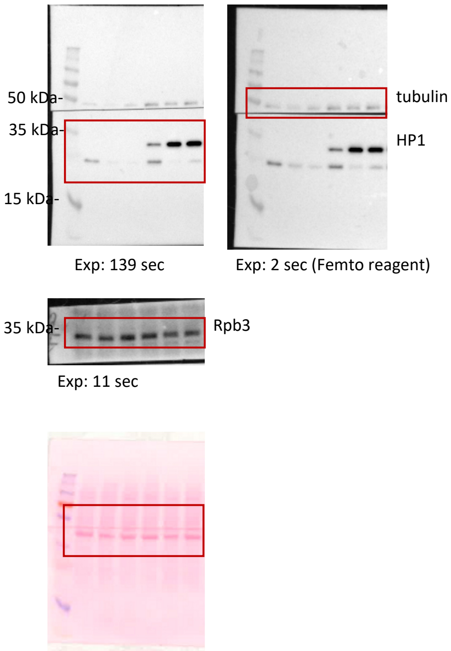

Extended Data Fig 4i

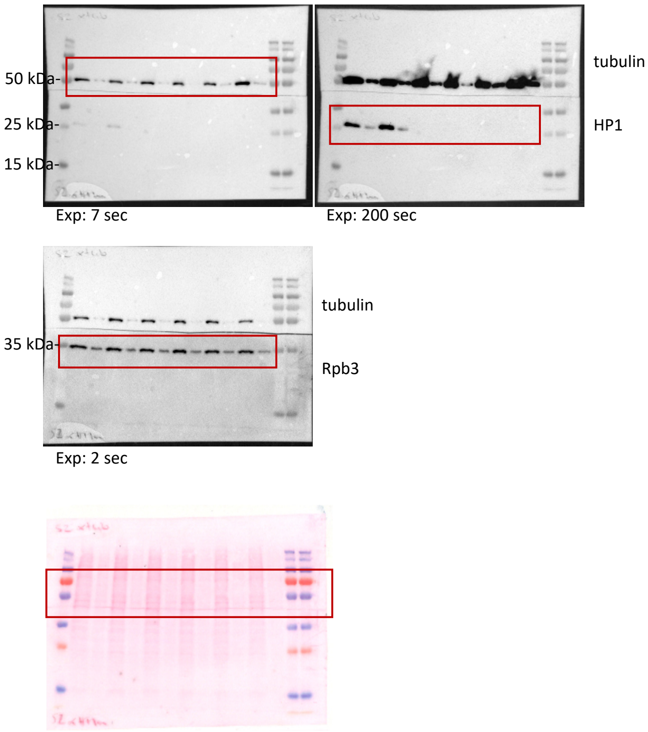

Supplement: Supplementary file 1 — This file contains all Materials and Methods as well as Supplementary Fig.1 with all raw images of the Western Blots performed in the study. [file 41586_2021_3460_MOESM1_ESM.pdf]
