# Supplementary material for: HP1 drives de novo 3D genome reorganization in early Drosophila embryos
Source: Nature. 2021 Apr 14;593(7858):289–93. doi: 10.1038/s41586-021-03460-z (PMC8116211; doi:10.1038/s41586-021-03460-z)
Supplement: Supplementary file 2 — Reporting Summary [file 41586_2021_3460_MOESM2_ESM.pdf]

nature research

Corresponding author(s): Luca Giorgetti, Nicola Iovino

Last updated by author(s): 02.03.2021

Reporting Summary

Nature Research wishes to improve the reproducibility of the work that we publish. This form provides structure for consistency and transparency in reporting. For further information on Nature Research policies, see our Editorial Policies and the Editorial Policy Checklist.

Statistics

For all statistical analyses, confirm that the following items are present in the figure legend, table legend, main text, or Methods section.

n/a	Confirmed	
☐	☒	The exact sample size (*n*) for each experimental group/condition, given as a discrete number and unit of measurement
☐	☒	A statement on whether measurements were taken from distinct samples or whether the same sample was measured repeatedly
☐	☒	The statistical test(s) used AND whether they are one- or two-sided<br>*Only common tests should be described solely by name; describe more complex techniques in the Methods section.*
☐	☒	A description of all covariates tested
☐	☒	A description of any assumptions or corrections, such as tests of normality and adjustment for multiple comparisons
☐	☒	A full description of the statistical parameters including central tendency (e.g. means) or other basic estimates (e.g. regression coefficient) AND variation (e.g. standard deviation) or associated estimates of uncertainty (e.g. confidence intervals)
☐	☒	For null hypothesis testing, the test statistic (e.g. *F*, *t*, *r*) with confidence intervals, effect sizes, degrees of freedom and *P* value noted<br>*Give P values as exact values whenever suitable.*
☐	☒	For Bayesian analysis, information on the choice of priors and Markov chain Monte Carlo settings
☒	☐	For hierarchical and complex designs, identification of the appropriate level for tests and full reporting of outcomes
☒	☐	Estimates of effect sizes (e.g. Cohen's *d*, Pearson's *r*), indicating how they were calculated

Our web collection on statistics for biologists contains articles on many of the points above.

Software and code

Policy information about availability of computer code

Data collection	Western Blots were imaged using ChemiDoc Imaging System with Image-Lab(Bio-Rad), qPCR data was collected using LightCycler 96 (Roche). For immunofluorescence Zen (Zeiss) was used to record the data.
Data analysis	QuasR v1.30, deepTools v2.4.2, MonteGrappa v1.2, HiC-Pro v2.11.1, HiTC v1.32.0, IDR v.2.0, reshape2 v1.4.4, rtracklayer v1.50.0, Rcpp v1.0.6, GenomicRanges v1.42.0, matrix2insulation.pl v1.0.0, MACS2 2.1.3.3, STAR v2.7.0, FIJI v.2.1.0, LAMMPS, GraphPad Prism 6, Imaris 9.5.1 (Bitplane) Further settings of the Hi-C, compartment analysis and the simulation have been made available here: https://github.com/zhanyinx/Zenk_Zhan_et_al_Nature2021

For manuscripts utilizing custom algorithms or software that are central to the research but not yet described in published literature, software must be made available to editors and reviewers. We strongly encourage code deposition in a community repository (e.g. GitHub). See the Nature Research guidelines for submitting code & software for further information.

Data

Policy information about availability of data

All manuscripts must include a data availability statement. This statement should provide the following information, where applicable:

- Accession codes, unique identifiers, or web links for publicly available datasets
- A list of figures that have associated raw data
- A description of any restrictions on data availability

All HiC, ChIP and RNA-Seq raw files generated in this study has been uploaded to GEO (GSE140542).
https://www.ncbi.nlm.nih.gov/geo/query/acc.cgi?acc=GSE140542

Also processed data are available there. The bed files of called HP1 peaks is provided as Supplementary Tables of this study. Further a table with RNA-Seq counts is

available as Supplementary Table.

Public databases used: BSgenome.Dmelanogaster.UCSC.dm6, org.Dm.eg.db, TxDb.Dmelanogaster.UCSC.dm6.ensGene

Field-specific reporting

Please select the one below that is the best fit for your research. If you are not sure, read the appropriate sections before making your selection.

☒ Life sciences ☐ Behavioural & social sciences ☐ Ecological, evolutionary & environmental sciences

For a reference copy of the document with all sections, see nature.com/documents/nr-reporting-summary-flat.pdf

Life sciences study design

All studies must disclose on these points even when the disclosure is negative.

Sample size	For Hi-C data we used 5-7 biological replicates. All ChIP-Seq experiments have been performed at least in biological replicates. For RNA-Seq 3-4 biological replicates were collected. We did not apply statistical methods to pre-determine sample size and followed the general standard practice in the field. Number of replicate experiments is indicated in the legends.
Data exclusions	We did not exclude data.
Replication	We performed all experiments in biological replicates and could observe agreement between the replicates. All experiments were performed at least twice independently and material was collected independently and by different researchers.
Randomization	We controlled variability by collecting the samples in several batches and by employing different researchers. Samples were allocated randomly to the researcher. We also performed a high number of biological replicates and collected pools of embryos at the same developmental stage.
Blinding	We did not perform blinded experiments. Complete blinding was not possible because the mutant phenotypes were evident from the development of the embryo. First computational analysis and inspection of HiC data were performed blinded.

Behavioural & social sciences study design

All studies must disclose on these points even when the disclosure is negative.

Study description	*Briefly describe the study type including whether data are quantitative, qualitative, or mixed-methods (e.g. qualitative cross-sectional, quantitative experimental, mixed-methods case study).*
Research sample	*State the research sample (e.g. Harvard university undergraduates, villagers in rural India) and provide relevant demographic information (e.g. age, sex) and indicate whether the sample is representative. Provide a rationale for the study sample chosen. For studies involving existing datasets, please describe the dataset and source.*
Sampling strategy	*Describe the sampling procedure (e.g. random, snowball, stratified, convenience). Describe the statistical methods that were used to predetermine sample size OR if no sample-size calculation was performed, describe how sample sizes were chosen and provide a rationale for why these sample sizes are sufficient. For qualitative data, please indicate whether data saturation was considered, and what criteria were used to decide that no further sampling was needed.*
Data collection	*Provide details about the data collection procedure, including the instruments or devices used to record the data (e.g. pen and paper, computer, eye tracker, video or audio equipment) whether anyone was present besides the participant(s) and the researcher, and whether the researcher was blind to experimental condition and/or the study hypothesis during data collection.*
Timing	*Indicate the start and stop dates of data collection. If there is a gap between collection periods, state the dates for each sample cohort.*
Data exclusions	*If no data were excluded from the analyses, state so OR if data were excluded, provide the exact number of exclusions and the rationale behind them, indicating whether exclusion criteria were pre-established.*
Non-participation	*State how many participants dropped out/declined participation and the reason(s) given OR provide response rate OR state that no participants dropped out/declined participation.*
Randomization	*If participants were not allocated into experimental groups, state so OR describe how participants were allocated to groups, and if allocation was not random, describe how covariates were controlled.*

Ecological, evolutionary & environmental sciences study design

All studies must disclose on these points even when the disclosure is negative.

Study description	*Briefly describe the study. For quantitative data include treatment factors and interactions, design structure (e.g. factorial, nested, hierarchical), nature and number of experimental units and replicates.*
Research sample	*Describe the research sample (e.g. a group of tagged Passer domesticus, all Stenocereus thurberi within Organ Pipe Cactus National Monument), and provide a rationale for the sample choice. When relevant, describe the organism taxa, source, sex, age range and any manipulations. State what population the sample is meant to represent when applicable. For studies involving existing datasets, describe the data and its source.*
Sampling strategy	*Note the sampling procedure. Describe the statistical methods that were used to predetermine sample size OR if no sample-size calculation was performed, describe how sample sizes were chosen and provide a rationale for why these sample sizes are sufficient.*
Data collection	*Describe the data collection procedure, including who recorded the data and how.*
Timing and spatial scale	*Indicate the start and stop dates of data collection, noting the frequency and periodicity of sampling and providing a rationale for these choices. If there is a gap between collection periods, state the dates for each sample cohort. Specify the spatial scale from which the data are taken*
Data exclusions	*If no data were excluded from the analyses, state so OR if data were excluded, describe the exclusions and the rationale behind them, indicating whether exclusion criteria were pre-established.*
Reproducibility	*Describe the measures taken to verify the reproducibility of experimental findings. For each experiment, note whether any attempts to repeat the experiment failed OR state that all attempts to repeat the experiment were successful.*
Randomization	*Describe how samples/organisms/participants were allocated into groups. If allocation was not random, describe how covariates were controlled. If this is not relevant to your study, explain why.*
Blinding	*Describe the extent of blinding used during data acquisition and analysis. If blinding was not possible, describe why OR explain why blinding was not relevant to your study.*

Did the study involve field work? ☐ Yes ☐ No

Field work, collection and transport

Field conditions	*Describe the study conditions for field work, providing relevant parameters (e.g. temperature, rainfall).*
Location	*State the location of the sampling or experiment, providing relevant parameters (e.g. latitude and longitude, elevation, water depth).*
Access & import/export	*Describe the efforts you have made to access habitats and to collect and import/export your samples in a responsible manner and in compliance with local, national and international laws, noting any permits that were obtained (give the name of the issuing authority, the date of issue, and any identifying information).*
Disturbance	*Describe any disturbance caused by the study and how it was minimized.*

Reporting for specific materials, systems and methods

We require information from authors about some types of materials, experimental systems and methods used in many studies. Here, indicate whether each material, system or method listed is relevant to your study. If you are not sure if a list item applies to your research, read the appropriate section before selecting a response.

Materials & experimental systems

n/a	Involved in the study	
☐	☒	Antibodies
☐	☒	Eukaryotic cell lines
☒	☐	Palaeontology and archaeology
☐	☒	Animals and other organisms
☒	☐	Human research participants
☒	☐	Clinical data
☒	☐	Dual use research of concern

Methods

n/a	Involved in the study	
☐	☒	ChIP-seq
☒	☐	Flow cytometry
☒	☐	MRI-based neuroimaging

Antibodies

Antibodies used

A detailed list of all antibodies used in the study is provided in the materials and methods.
HP1 Developmental Studies Hybridoma Bank C1A9
HP1 Covance, PRB-291C-200
HA (C29F4) Cell Signaling #3724S
HA.11 Covance MMS-101P
H3K9ac ActiveMotif 39586
H3K9ac ActiveMotif 39918
H3K9me3 ActiveMotif 39161
H3K9me2 ActiveMotif 39239
H3K9me3 Abcam ab176916
H3K27me3 Diagenode, C15410195 (Lot A1811-001P)
H3K4me1 Diagenode, C15410194 (Lot A1862D)
H3K4me3 Diagenode, C15410003
H3K27ac Diagenode, C15410196 (Lot A1723-041D)
Rpb3 (PolII) Carla Margulies Lab
Tubulin DM1 Sigma T9026
H3 ActiveMotif MABI 0301
Rpb3 (PolII) Asifa Akhtar Lab

Validation

Antibodies used in this study are commercially available and have been validated by the manufacturer. We further validated antibodies against H3K9me3, H3K9me2, H3K9ac, H3K27me3, H3K27ac, H3K4me1, HP1, Rpb3 either by Immunofluorescence staining or Western Blot in the control and the knockdown of the respective epigenetic writer or the protein itself.
HP1 Developmental Studies Hybridoma Bank C1A9 (validated by western blot and ChIP in HP1-KD in ED Fig. 1g and 3b)
HP1 Covance, PRB-291C-200
HA (C29F4) Cell Signaling #3724S (according to manufacturer used in 866 oublications, validated for ChIP)
HA.11 Covance MMS-101P
H3K9ac ActiveMotif 39586 (according to manufacturer used in 7 publications, validated for ChIP)
H3K9ac ActiveMotif 39918 (according to manufacturer validated for ChIP and NGS applications)
H3K9me3 ActiveMotif 39161 (according to manufacturer validated for ChIP and NGS applications)
H3K9me2 ActiveMotif 39239 (validated by immunofluorescence in K9M in ED Fig. 4a)
H3K9me3 Abcam ab176916 (according to manufacturer used in 11 publications, validated for ChIP)
H3K27me3 Diagenode, C15410195 (Lot A1811-001P) (validated by ChIP in E(z)-KD in Zenk et al. 2017, Science)
H3K4me1 Diagenode, C15410194 (Lot A1862D) (according to manufacturer used in 44 publications, validated for ChIP)
H3K4me3 Diagenode, C15410003 (according to manufacturer used in 184 publications, validated for ChIP)
H3K27ac Diagenode, C15410196 (Lot A1723-041D) (according to manufacturer validated for ChIP and NGS applications)
Rpb3 (PolII) Carla Margulies Lab
Tubulin DM1 Sigma T9026
H3 ActiveMotif MABI 0301
Rpb3 (PolII) Asifa Akhtar Lab (validated by western blot in Rpb3 KD embryos)

Eukaryotic cell lines

Policy information about cell lines

Cell line source(s)

We used S2 cell line provided by Dr. Michael Boutros, DKFZ, Heidelberg. Details about the cell line are included in the materials and methods. Origin of S2 cells Schneider I (1972). "Cell Lines Derived from Late Embryonic Stages of Drosophila melanogaster". J. Embryol. Exp. Morphol. 27: 363–365.

Authentication

The cell line has been authenticated by visual inspection of the morphology. Further total RNA-Sequencing indicated absence of viral or microbial infections.

Mycoplasma contamination

The cell line tested negative for Mycoplasma.

Commonly misidentified lines
(See ICLAC register)

No commonly misidentified cell lines were used in the study.

Palaeontology and Archaeology

Specimen provenance

Provide provenance information for specimens and describe permits that were obtained for the work (including the name of the issuing authority, the date of issue, and any identifying information).

Specimen deposition

Indicate where the specimens have been deposited to permit free access by other researchers.

Dating methods

If new dates are provided, describe how they were obtained (e.g. collection, storage, sample pretreatment and measurement), where they were obtained (i.e. lab name), the calibration program and the protocol for quality assurance OR state that no new dates are provided.

☐ Tick this box to confirm that the raw and calibrated dates are available in the paper or in Supplementary Information.

Ethics oversight

Identify the organization(s) that approved or provided guidance on the study protocol, OR state that no ethical approval or guidance

Ethics oversight	*was required and explain why not.*

Note that full information on the approval of the study protocol must also be provided in the manuscript.

Animals and other organisms

Policy information about studies involving animals; ARRIVE guidelines recommended for reporting animal research

Laboratory animals	We used Drosophila melanogaster embryos to perform all experiments presented. We used fly lines encoding short hairpin RNAs against the target provided by the TRiP consortium and made available through the Bloomington stock center (Driver #7063, HP1-KD #33400). A description of fly lines generated in this study is provided in the materials and methods. All work was conducted in Drosophila melanogaster, an invertebrate animal (hexapod arthropod from the insect group). Invertebrate models are not regulated by the TierSchVersV and therefore are not subjected to ethical approval.
Wild animals	The study did not involve wild animals.
Field-collected samples	The study did not involve samples collected from the field.
Ethics oversight	All work was conducted in Drosophila melanogaster, an invertebrate animal (hexapod arthropod from the insect group). Invertebrate models are not regulated by the TierSchVersV and therefore are not subjected to ethical approval.

Note that full information on the approval of the study protocol must also be provided in the manuscript.

Human research participants

Policy information about studies involving human research participants

Population characteristics	*Describe the covariate-relevant population characteristics of the human research participants (e.g. age, gender, genotypic information, past and current diagnosis and treatment categories). If you filled out the behavioural & social sciences study design questions and have nothing to add here, write "See above."*
Recruitment	*Describe how participants were recruited. Outline any potential self-selection bias or other biases that may be present and how these are likely to impact results.*
Ethics oversight	*Identify the organization(s) that approved the study protocol.*

Note that full information on the approval of the study protocol must also be provided in the manuscript.

Clinical data

Policy information about clinical studies

All manuscripts should comply with the ICMJE guidelines for publication of clinical research and a completed CONSORT checklist must be included with all submissions.

Clinical trial registration	*Provide the trial registration number from ClinicalTrials.gov or an equivalent agency.*
Study protocol	*Note where the full trial protocol can be accessed OR if not available, explain why.*
Data collection	*Describe the settings and locales of data collection, noting the time periods of recruitment and data collection.*
Outcomes	*Describe how you pre-defined primary and secondary outcome measures and how you assessed these measures.*

Dual use research of concern

Policy information about dual use research of concern

Hazards

Could the accidental, deliberate or reckless misuse of agents or technologies generated in the work, or the application of information presented in the manuscript, pose a threat to:

No	Yes	
☐	☐	Public health
☐	☐	National security
☐	☐	Crops and/or livestock
☐	☐	Ecosystems
☐	☐	Any other significant area

Experiments of concern

Does the work involve any of these experiments of concern:

No	Yes	
☐	☐	Demonstrate how to render a vaccine ineffective
☐	☐	Confer resistance to therapeutically useful antibiotics or antiviral agents
☐	☐	Enhance the virulence of a pathogen or render a nonpathogen virulent
☐	☐	Increase transmissibility of a pathogen
☐	☐	Alter the host range of a pathogen
☐	☐	Enable evasion of diagnostic/detection modalities
☐	☐	Enable the weaponization of a biological agent or toxin
☐	☐	Any other potentially harmful combination of experiments and agents

ChIP-seq

Data deposition

☒ Confirm that both raw and final processed data have been deposited in a public database such as GEO.

☒ Confirm that you have deposited or provided access to graph files (e.g. BED files) for the called peaks.

Data access links
May remain private before publication.

All HiC, ChIP and RNA-Seq raw files generated in this study has been uploaded to GEO (GSE140542). https://www.ncbi.nlm.nih.gov/geo/query/acc.cgi?acc=GSE140542

Files in database submission

GSM4173897 ChIP H3K27ac IP repl1
GSM4173898 ChIP H3K27ac, H3K4me1 and H3K4me3 input repl1
GSM4173899 ChIP H3K27ac IP repl2
GSM4173900 ChIP H3K27ac and H3K4me3 input repl2
GSM4173901 ChIP H3K27me3 IP repl1
GSM4173902 ChIP H3K27me3 and Pol2 input repl1
GSM4173903 ChIP H3K27me3 IP repl2
GSM4173904 ChIP H3K27me3 and Pol2 input repl2
GSM4173905 ChIP H3K4me1 IP repl2
GSM4173906 ChIP H3K4me1 input repl2
GSM4173907 ChIP H3K4me1 IP repl1
GSM4173908 ChIP H3K4me3 IP repl1
GSM4173909 ChIP H3K4me3 IP repl2
GSM4173910 ChIP H3K9ac input repl1
GSM4173911 ChIP H3K9ac IP repl1
GSM4173912 ChIP H3K9ac IP repl2
GSM4173913 ChIP H3K9me3 IP repl1
GSM4173914 ChIP H3K9me3 input repl1
GSM4173915 ChIP H3K9me3 IP repl2
GSM4173916 ChIP H3K9me3 input repl2
GSM4173917 ChIP HP1 IP repl1
GSM4173918 ChIP HP1 input repl1
GSM4173919 ChIP HP1 input repl2
GSM4173920 ChIP HP1 IP repl2
GSM4173921 ChIP HP1 input repl3
GSM4173922 ChIP HP1 IP repl3
GSM4173923 ChIP HP1 IP repl1 Flag HA
GSM4173924 ChIP HP1 input repl1 Flag HA
GSM4173925 ChIP HP1 IP repl1 DSG
GSM4173926 ChIP HP1 input repl1 DSG
GSM4173927 ChIP Pol2 IP repl1
GSM4173928 ChIP Pol2 IP repl2
GSM4173929 HiC WT stage 5 repl1
GSM4173930 HiC WT stage 5 repl2
GSM4173931 HiC WT stage 5 repl3
GSM4173932 HiC WT stage 5 repl4
GSM4173933 HiC WT stage 5 repl5
GSM4173934 HiC WT stage 5 repl6
GSM4173935 HiC WT stage 5 repl7
GSM4173936 HiC HP1 KD stage 5 repl 1
GSM4173938 HiC HP1 KD stage 5 repl 3
GSM4173939 HiC HP1 KD stage 5 repl 4
GSM4173940 HiC HP1 KD stage 5 repl 5
GSM4173941 HiC HP1 KD + rescued stage 5 repl 1
GSM4173942 HiC HP1 KD + rescued stage 5 repl 2

GSM4173943 HiC HP1 KD + rescued stage 5 repl 3
GSM4173944 HiC K9M stage 5 repl1
GSM4173946 RNA-seq WT repl1
GSM4173947 RNA-seq WT repl2
GSM4173948 RNA-seq WT repl3
GSM4173949 RNA-seq WT repl4
GSM4173950 RNA-seq HP1 KD repl1
GSM4173951 RNA-seq HP1 KD repl2
GSM4173952 RNA-seq HP1 KD repl3
GSM4576008 HiC HP1 KD stage 5 repl 2
GSM4576009 HiC K9M stage 5 repl2
GSM4594789 HiC WT S2 cells repl 1
GSM4594790 HiC WT S2 cells repl 2
GSM4594791 HiC HP1 KD rnai1 S2 cells repl 1
GSM4594792 HiC HP1 KD rnai1 S2 cells repl 2
GSM4594793 HiC HP1 KD rnai2 S2 cells repl 1
GSM4594794 HiC HP1 KD rnai2 S2 cells repl 2
GSM4595523 ChIP HP1 IP repl1 Covance
GSM4595524 ChIP HP1 Input repl1 Covance
GSM4595525 ChIP HP1 IP repl2 Covance
GSM4595526 ChIP HP1 Input repl2 Covance
GSM4595527 ChIP HP1 Input repl1 before cycle 9 with spike in
GSM4595528 ChIP HP1 IP repl1 before cycle 9 with spike in
GSM4595529 ChIP HP1 Input repl2 before cycle 9 with spike in
GSM4595530 ChIP HP1 IP repl2 before cycle 9 with spike in
GSM4595531 ChIP HP1 Input repl1 cycle 9-13 with spike in
GSM4595532 ChIP HP1 IP repl1 cycle 9-13 with spike in
GSM4595533 ChIP HP1 Input repl2 cycle 9-13 with spike in
GSM4595534 ChIP HP1 IP repl2 cycle 9-13 with spike in
GSM4595535 ChIP HP1 Input repl1 stg5 HP1KD embryo with spike in
GSM4595536 ChIP HP1 IP repl1 stg5 HP1KD embryo with spike in
GSM4595537 ChIP HP1 Input repl2 stg5 HP1KD embryo with spike in
GSM4595538 ChIP HP1 IP repl2 stg5 HP1KD embryo with spike in
GSM4595539 ChIP HP1 Input repl1 stg5 K9M embryo with spike in
GSM4595540 ChIP HP1 IP repl1 stg5 K9M embryo with spike in
GSM4595541 ChIP HP1 Input repl2 stg5 K9M embryo with spike in
GSM4595542 ChIP HP1 IP repl2 stg5 K9M embryo with spike in
GSM4595543 ChIP HP1 Input repl1 stg5 WT embryo with spike in
GSM4595544 ChIP HP1 IP repl1 stg5 WT embryo with spike in
GSM4595545 ChIP HP1 Input repl2 stg5 WT embryo with spike in
GSM4595546 ChIP HP1 IP repl2 stg5 WT embryo with spike in
GSM4983387 ChIP HP1 IP repl1 in ovaries
GSM4983388 ChIP HP1 Input repl1 in ovaries
GSM4983389 ChIP HP1-CD IP repl1 in ovaries
GSM4983390 ChIP HP1-CD Input repl1 in ovaries
GSM4983391 ChIP HP1 IP repl2 in ovaries
GSM4983392 ChIP HP1 Input repl2 in ovaries
GSM4983393 ChIP HP1-CD IP repl2 in ovaries
GSM4983394 ChIP HP1-CD Input repl2 in ovaries

Genome browser session (e.g. UCSC)

All bigwig files are available in GEO (GSE140542).

Methodology

Replicates

All ChIP experiments were performed in biological replicates. We inspected the agreement of replicates visually in the genome browser and by computing heatmaps on the individual replicates.

Sequencing depth

We sequenced all samples in the study at least to a depth of 15 Mio.

Antibodies

A detailed list of all antibodies used in the study is provided in the materials and methods (table 1). The HP1 antibody was also validated by performing ChIP-seq in HP1-KD embryos.

Peak calling parameters

Peaks were called using macs2, using a cut-off of 0.05 on the q-value. --broad option was added for the H3K9me3 and HP1 ChIP. Further details are available in the materials and methods.

Data quality

We visually inspected all ChIP tracks and called peaks in the genome browser. To call the peaks we used a cut-off of 0.05 on the q-value and only included peaks that passed this threshold.

Software

Reads were mapped to the D. melanogaster genome (build dm6) using qAlign from QuasR package using the following alignments parameters: -n 2 -l 28 -e 70 -k 1 -X 500. This allows to keep reads mapping to multiple loci and randomly assigns them to one of the multiple locations. ChIP enrichment over input was calculated using bamCompare from deepTools using the following parameters: --scaleFactorsMethod SES --smoothLength 900 --binSize 300. Further details are available in the materials and methods.

Flow Cytometry

Plots

Confirm that:

- [ ] The axis labels state the marker and fluorochrome used (e.g. CD4-FITC).
- [ ] The axis scales are clearly visible. Include numbers along axes only for bottom left plot of group (a 'group' is an analysis of identical markers).
- [ ] All plots are contour plots with outliers or pseudocolor plots.
- [ ] A numerical value for number of cells or percentage (with statistics) is provided.

Methodology

Sample preparation	*Describe the sample preparation, detailing the biological source of the cells and any tissue processing steps used.*
Instrument	*Identify the instrument used for data collection, specifying make and model number.*
Software	*Describe the software used to collect and analyze the flow cytometry data. For custom code that has been deposited into a community repository, provide accession details.*
Cell population abundance	*Describe the abundance of the relevant cell populations within post-sort fractions, providing details on the purity of the samples and how it was determined.*
Gating strategy	*Describe the gating strategy used for all relevant experiments, specifying the preliminary FSC/SSC gates of the starting cell population, indicating where boundaries between "positive" and "negative" staining cell populations are defined.*

- [ ] Tick this box to confirm that a figure exemplifying the gating strategy is provided in the Supplementary Information.

Magnetic resonance imaging

Experimental design

Design type	*Indicate task or resting state; event-related or block design.*
Design specifications	*Specify the number of blocks, trials or experimental units per session and/or subject, and specify the length of each trial or block (if trials are blocked) and interval between trials.*
Behavioral performance measures	*State number and/or type of variables recorded (e.g. correct button press, response time) and what statistics were used to establish that the subjects were performing the task as expected (e.g. mean, range, and/or standard deviation across subjects).*

Acquisition

Imaging type(s)	*Specify: functional, structural, diffusion, perfusion.*
Field strength	*Specify in Tesla*
Sequence & imaging parameters	*Specify the pulse sequence type (gradient echo, spin echo, etc.), imaging type (EPI, spiral, etc.), field of view, matrix size, slice thickness, orientation and TE/TR/flip angle.*
Area of acquisition	*State whether a whole brain scan was used OR define the area of acquisition, describing how the region was determined.*

Diffusion MRI ☐ Used ☐ Not used

Preprocessing

Preprocessing software	*Provide detail on software version and revision number and on specific parameters (model/functions, brain extraction, segmentation, smoothing kernel size, etc.).*
Normalization	*If data were normalized/standardized, describe the approach(es): specify linear or non-linear and define image types used for transformation OR indicate that data were not normalized and explain rationale for lack of normalization.*
Normalization template	*Describe the template used for normalization/transformation, specifying subject space or group standardized space (e.g. original Talairach, MNI305, ICBM152) OR indicate that the data were not normalized.*
Noise and artifact removal	*Describe your procedure(s) for artifact and structured noise removal, specifying motion parameters, tissue signals and physiological signals (heart rate, respiration).*

Volume censoring | *Define your software and/or method and criteria for volume censoring, and state the extent of such censoring.*

Statistical modeling & inference

Model type and settings | *Specify type (mass univariate, multivariate, RSA, predictive, etc.) and describe essential details of the model at the first and second levels (e.g. fixed, random or mixed effects; drift or auto-correlation).*

Effect(s) tested | *Define precise effect in terms of the task or stimulus conditions instead of psychological concepts and indicate whether ANOVA or factorial designs were used.*

Specify type of analysis: ☐ Whole brain ☐ ROI-based ☐ Both

Statistic type for inference (See Eklund et al. 2016) | *Specify voxel-wise or cluster-wise and report all relevant parameters for cluster-wise methods.*

Correction | *Describe the type of correction and how it is obtained for multiple comparisons (e.g. FWE, FDR, permutation or Monte Carlo).*

Models & analysis

n/a	Involved in the study
☐	☐ Functional and/or effective connectivity
☐	☐ Graph analysis
☐	☐ Multivariate modeling or predictive analysis

Functional and/or effective connectivity | *Report the measures of dependence used and the model details (e.g. Pearson correlation, partial correlation, mutual information).*

Graph analysis | *Report the dependent variable and connectivity measure, specifying weighted graph or binarized graph, subject- or group-level, and the global and/or node summaries used (e.g. clustering coefficient, efficiency, etc.).*

Multivariate modeling and predictive analysis | *Specify independent variables, features extraction and dimension reduction, model, training and evaluation metrics.*
